# Identification of PANoptosis hub genes driving immune activation and tubulointerstitial injury in diabetic kidney disease by integrative bioinformatics and machine learning

**DOI:** 10.3389/fimmu.2026.1759781

**Published:** 2026-03-09

**Authors:** Yintong Chen, Feifei Yuan, Shengyuan Li, Lerong Liu, Xuankun Peng, Xiangrong Zeng, Siyong Chen, Nianping Liu, Tongfeng Zhao

**Affiliations:** 1 Department of Geriatric Endocrinology, Guangdong Geriatrics Institute, Guangdong Provincial People’s Hospital (Guangdong Academy of Medical Sciences), Southern Medical University, Guangzhou, China; 2 Division of Nephrology, Nanfang Hospital, Southern Medical University, Guangzhou, China; 3 Department of Endocrinology and Metabolism, Nanfang Hospital, Southern Medical University, Guangzhou, China; 4 Department of Endocrinology, The Sixth Affiliated Hospital, Sun Yat-sen University, Guangzhou, China; 5 The First Affiliated Hospital of University of Science and Technology of China (USTC), School of Basic Medical Sciences, Division of Life Sciences and Medicine, University of Science and Technology of China, Hefei, China

**Keywords:** diabetic kidney disease, DrugnomeAI, immune infiltration, machine learning, PANoptosis, tubules and tubulointerstitium

## Abstract

**Background:**

Diabetic kidney disease (DKD) is characterized by chronic inflammation and immune dysregulation. Multiple programmed cell death pathways contribute to tubulointerstitial injury, but their perturbations, crosstalk, and integrative impact in DKD remain unclear. PANoptosis—a coordinated program integrating pyroptosis, apoptosis, and necroptosis—has emerged as a key mechanism in inflammatory disorders, yet its role in DKD is not defined.

**Methods:**

We integrated multiple renal tubulointerstitial transcriptomic datasets from DKD and control cohorts to identify differentially expressed genes, followed by functional enrichment analysis. PANoptosis-related gene sets were curated from MSigDB, and pathway crosstalk was evaluated using independent single-cell RNA-seq datasets. Hub genes were prioritized by combining weighted gene co-expression network analysis (WGCNA) with five machine-learning algorithms, and a PANoptosis-related risk score (PRS) was constructed and correlated with clinical parameters and immune infiltration. miRNA–mRNA and transcription factor–hub gene regulatory networks were inferred using ENCORI and hTFtarget, respectively. Druggability of hub genes was assessed using DrugnomeAI, and candidate compounds were retrieved from DGIdb. Key findings were validated in diabetic mouse models.

**Results:**

Apoptosis, pyroptosis, necroptosis and the integrated PANoptosis program were markedly activated in DKD. At the single-cell level, these pathways were frequently co-activated within tubular and interstitial cell types, with extensive molecular overlap. Six PANoptosis-related hub genes (*YWHAH, PRKACB, PSMB9, FAS, GZMA, CASP1*) were identified; their expression correlated negatively with glomerular filtration rate and positively with serum creatinine and immune-cell infiltration. The PRS robustly discriminated DKD from controls and identified a high-risk subgroup with heightened immune infiltration and impaired renal function. Regulatory network analysis revealed convergent miRNA and transcription factor control of key hub genes. Druggability profiling with DrugnomeAI highlighted *CASP1, FAS, PSMB9* and *PRKACB* as experimentally tractable and pharmacologically actionable targets, and DGIdb suggested multiple repurposable agents against these nodes.

**Conclusion:**

This study delineates extensive perturbations and crosstalk among apoptosis, pyroptosis and necroptosis in DKD, positioning PANoptosis as a unifying driver of tubulointerstitial injury. The six PANoptosis hub genes and their derived PRS show strong diagnostic potential, while integrated regulatory and druggability analyses nominate *CASP1, FAS, PSMB9* and *PRKACB* as promising biomarkers and therapeutic entry points for PANoptosis-centered interventions in DKD.

## 1 Introduction

Diabetic kidney disease (DKD) is the leading global cause of end-stage renal disease (ESRD) (1). As diabetes prevalence rises, DKD-related ESRD already accounts for ~50% of cases in many developed countries, with 10-year mortality >30% (2). Early DKD is often clinically silent, and conventional indicators—albuminuria, creatinine, eGFR, and imaging—lack sensitivity at onset (3–5). Although renal biopsy remains the diagnostic gold standard, its invasiveness limits routine and longitudinal use (6). These gaps underscore the need for non-invasive biomarkers grounded in disease mechanisms, particularly the immune-inflammatory and programmed cell-death pathways now recognized as key drivers of DKD progression.

The pathogenesis of DKD is complex and multifactorial, with the traditional “glomerulus-centric” paradigm having dominated the field for decades (7). However, accumulating evidence suggests that damage to the renal tubules and tubulointerstitium may precede glomerular injury (8). Tubular structures are no longer seen as passive bystanders but are increasingly recognized as active contributors-and potentially key drivers-of DKD progression. Mechanistically, sustained metabolic disturbances, including hyperglycemia, dyslipidemia, and accumulation of advanced glycation end-products (AGEs), along with hemodynamic stress, hypoxia, and oxidative stress, collectively contribute to tubular damage (8, 9). This injury, in turn, triggers chronic inflammation and aberrant immune activation.

Unlike classical inflammatory diseases such as rheumatoid arthritis and sepsis, which are marked by acute cytokine storms, the inflammatory response in DKD is more subtle yet persistent, a phenomenon often described as “microinflammation” (10). This chronic inflammatory state is characterized by the upregulation of inflammatory signaling pathways, increased production of cytokines and chemokines, and infiltration of immune cells (11). Toll-like receptors (TLRs) recognize endogenous danger-associated molecular patterns (DAMPs) and initiate sterile tubulointerstitial inflammation via NF-κB signaling (12). The NLRP3 inflammasome promotes inflammatory cascades and fibrosis by inducing IL-1β and IL-18 (13). Similarly, the RIPK1-RIPK3 necrosome links regulated cell death to inflammation through MLKL activation (14). This evidence suggests that programmed cell death (PCD) serves as a key molecular link connecting tissue injury, inflammation, and immune activation.

PANoptosis is a newly defined form of inflammatory programmed cell death that integrates pyroptosis, apoptosis, and necroptosis through the assembly of a multiprotein complex known as the PANoptosome (15). This pathway enables coordinated, highly inflammatory cell death in response to specific triggers and represents a critical link between innate immunity and tissue injury. Unlike traditional PCD pathways, PANoptosis reflects the functional redundancy and crosstalk among multiple death mechanisms. A recent elegant study using single-cell RNA sequencing and gene knockout models demonstrated that PANoptosis plays a critical role in podocyte injury in diabetic kidney disease (16). However, under diabetic conditions, the specific roles of PANoptosis in tubular epithelial cells and the renal interstitium remain poorly understood and require further investigation.

**Figure 1:**
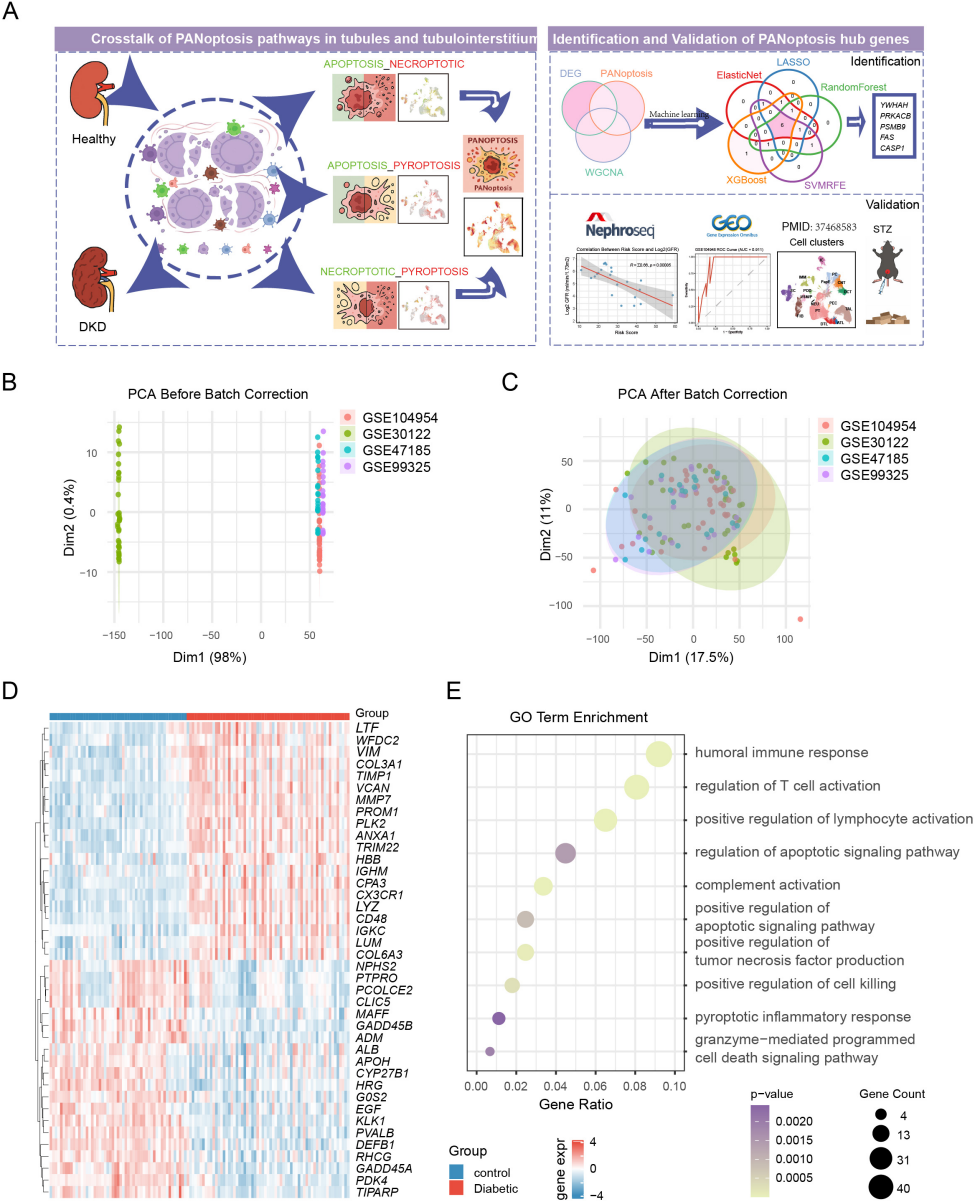
Identification of differentially expressed genes and enriched pathways in the tubulointerstitial tissue of patients with DN. **(A)** The overall workflow. **(B, C)** PCA plots before and after batch effect correction across four GEO datasets. **(D)** Heatmap of the top 20 most significantly dysregulated genes, showing distinct expression profiles between control and DKD samples. **(E)** GO enrichment analyses of DEGs, highlighting immune activation and cell death–related pathways.

In this study, we systematically analyzed the perturbations and crosstalk among pyroptosis, apoptosis, and necroptosis, providing robust evidence through both pathway enrichment analysis and cell-level visualization. PANoptosis-related genes were identified through comprehensive bioinformatics analyses, including weighted gene co-expression network analysis (WGCNA) and multiple machine learning algorithms, and a composite PANoptosis-related risk score (PRS) was constructed based on these key genes. The robustness of our findings was validated using independent datasets, single-cell RNA sequencing, and DKD mouse models. Furthermore, integrative regulatory and druggability analyses using DrugnomeAI and DGIdb highlighted CASP1, FAS, PSMB9, and PRKACB as central, experimentally tractable and pharmacologically actionable PANoptosis nodes, providing a rationale for biomarker development and therapeutic targeting in DKD. The overall workflow is illustrated in **Figure 1A**.

## 2 Results

### 2.1 Identification of differentially expressed genes and enrichment analysis between control and DKD

To investigate the role of the renal tubules and tubulointerstitium in the progression of DKD, we obtained four related datasets (GSE104954, GSE30122, GSE47185, and GSE99325) from the GEO database and merged them after correcting batch effects using the SVA algorithm. We validated the integration using PCA before and after correction (**Figures 1B, C**) and boxplot distributions (**Supplementary Figures 1A, B**). The analysis clearly separated control and DKD samples and identified 456 differentially expressed genes (DEGs), including 288 upregulated and 168 downregulated genes (**Supplementary Figure 1C**). The top 20 most significantly dysregulated genes were visualized in a heatmap (**Figure 1D**), showing distinct expression profiles between the two groups.

We then conducted GO, KEGG, and Reactome enrichment analyses using the “clusterProfiler” R package. GO analysis revealed significant enrichment in immune-related and pro-inflammatory processes, including humoral immune response, T cell activation, lymphocyte activation, and complement activation (**Figure 1E**). KEGG and Reactome analyses consistently highlighted inflammatory and immune pathways—such as T cell differentiation, NF-κB and chemokine signaling, cytokine networks, and complement cascades—emphasizing the contribution of immune dysregulation to DKD pathogenesis (**Supplementary Figures 1D, E**). In addition, pathways associated with programmed cell death, including apoptosis, pyroptosis, and necroptosis, were markedly enriched in DKD. Together, these findings underscore the central role of immune activation and inflammatory cell death in driving DKD progression.

### 2.2 Perturbations and crosstalk in PANoptosis in DKD

**Figure 2:**
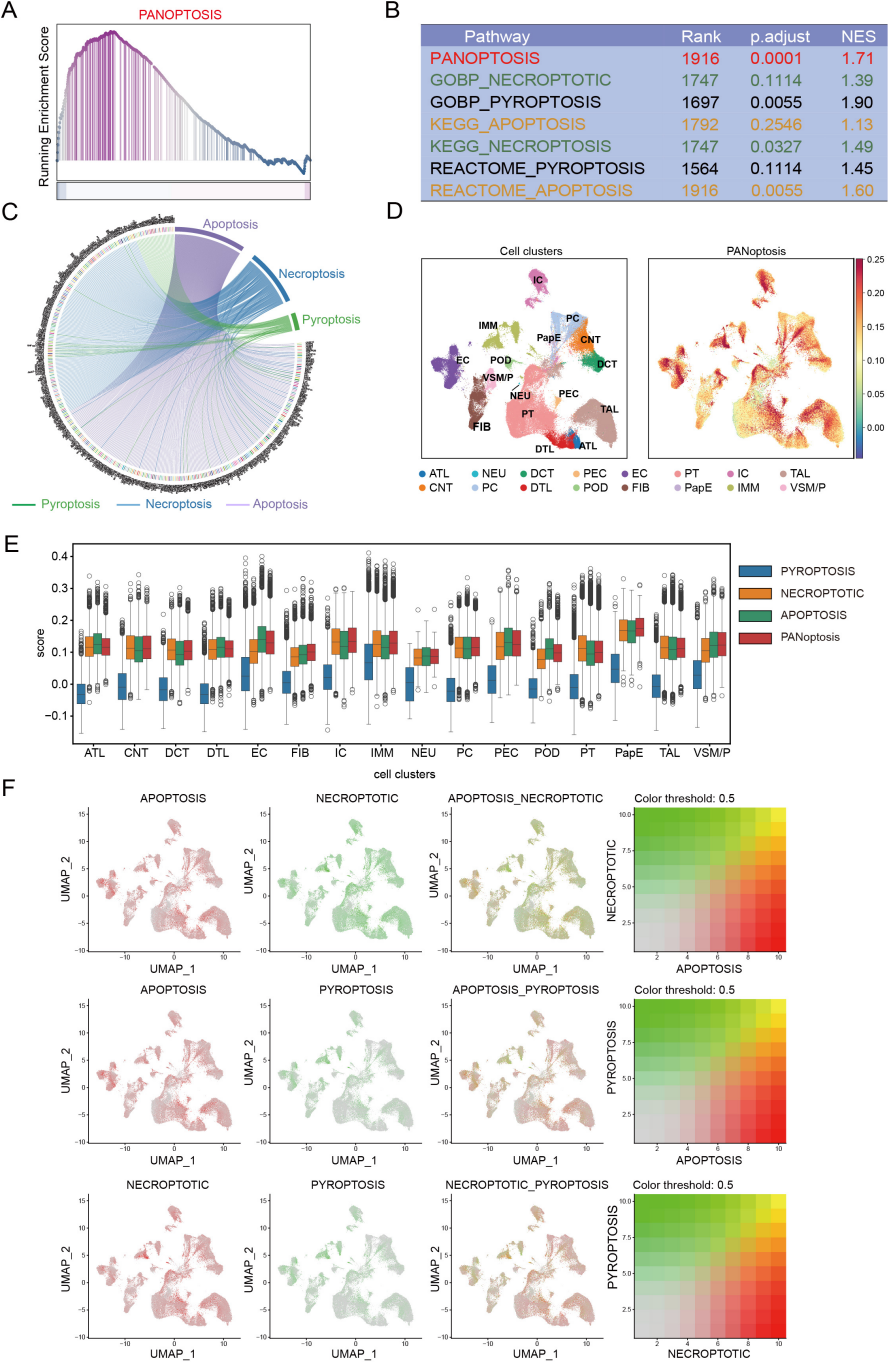
PANoptosis is broadly present across diverse cell types in DKD. **(A)** GSEA analysis showing significant enrichment of the integrated PANoptosis pathway in DKD. **(B)** Summary of GSEA results for apoptosis, pyroptosis, necroptosis, and PANoptosis signatures derived from the GO, KEGG, and Reactome databases. p.adjust, adjusted p-value; NES, normalized enrichment score. **(C)** Chord diagram illustrating gene-level overlap and crosstalk among the three programmed cell death pathways. Genes are displayed along the outer circle (**Supplementary Table 2**), and ribbons (chords) indicate genes shared between pathways. Ribbon colors denote the pathway category: pyroptosis (green), necroptosis (blue), and apoptosis (purple) (as indicated by the colored line legend). **(D)** Uniform Manifold Approximation and Projection (UMAP) of single-cell transcriptomes from DKD and healthy controls, colored by cell types (left) and PANoptosis scores (right). **(E)** Violin plots showing the distribution of apoptosis, pyroptosis, necroptosis, and PANoptosis signature scores across different cell types. **(F)** UMAP projections of paired programmed cell death scores at the single-cell level. For each pathway pair, the left and middle panels show the two individual signature scores, and the third panel shows a bivariate (combined) visualization of the two pathway scores. The corresponding bivariate color key is shown on the right, where the x- and y-axes represent the two pathway scores (e.g., apoptosis vs. necroptosis; apoptosis vs. pyroptosis; necroptosis vs. pyroptosis). Color threshold = 0.5 indicates the cutoff used to emphasize cells with relatively high co-activation within each pathway pair.

PANoptosis is a newly defined inflammatory form of programmed cell death that integrates three canonical pathways—apoptosis, pyroptosis, and necroptosis (17). To further elucidate its role in DKD, we systematically assessed the enrichment and interactions of these death programs. Gene set enrichment analysis (GSEA) was performed using curated gene sets from the GO, KEGG, and Reactome databases (**Supplementary Table 2**). All three pathways were significantly enriched in DKD samples compared with controls, with the integrated PANoptosis signature showing the strongest enrichment (adjusted p = 0.0001; **Figures 2A, B**; **Supplementary Figure 2A**). Pathway overlap analysis identified 61 genes shared by at least two pathways and 7 genes common to all three (**Figure 2C**, **Supplementary Figure 2B**), indicating substantial molecular crosstalk.

Next, we analyzed single-cell transcriptomic datasets of DKD and healthy controls obtained from public databases (18). PANoptosis was broadly detected across diverse cell types, including immune cells, renal tubular cells, and tubulointerstitial cells (**Figure 2D**). Among the three pathways, pyroptosis exhibited relatively lower activity across most cell types compared with necroptosis and apoptosis, while immune cells showed the highest levels of pyroptosis (**Figure 2E**). In addition, we performed subtype-level analysis of proximal tubule cells and immune populations (**Supplementary Figures 3A, B**). PANoptosis scores were highest in dendritic cells (DCs), natural killer cells (NK cells), and monocytes/macrophages (Mono/Mac) subsets, whereas PANoptosis scores were comparable across proximal tubule segments (**Supplementary Figures 3A, B**). Notably, individual cell types often displayed the coexistence of multiple forms of programmed cell death (**Figure 2F**), underscoring the complexity of PANoptosis regulation in DKD. Collectively, these results highlight PANoptosis as an integrated and highly interconnected cell death program contributing to DKD pathogenesis.

### 2.3 Identification of key co-expression module and PANoptosis-related DEGs via WGCNA

**Figure 3:**
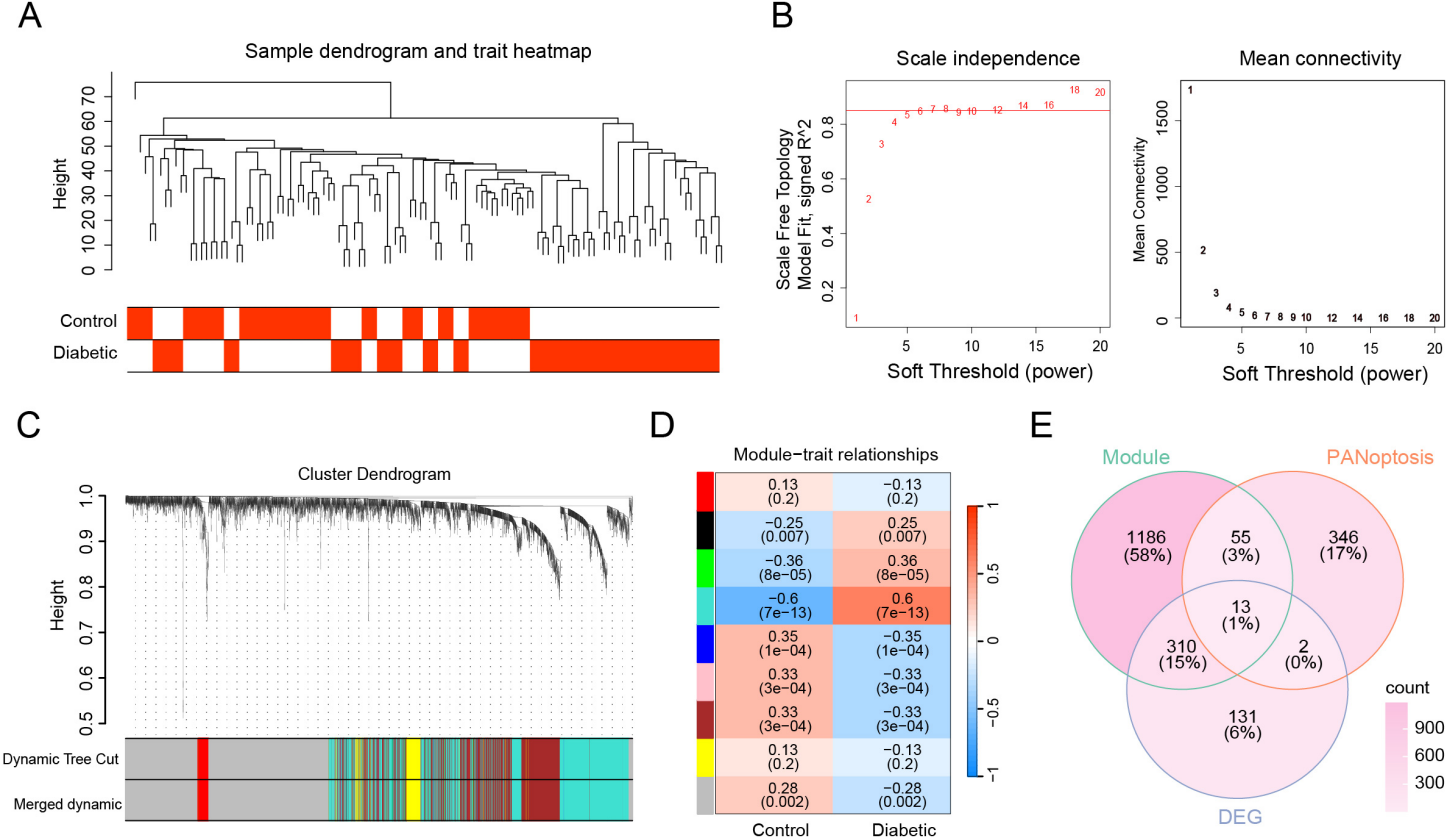
Construction of weighted gene co-expression network and identification of PANoptosis-related modules. **(A)** Sample clustering. To identify and remove outliers. **(B)** Analysis of scale independence and mean connectivity to determine the optimal soft-thresholding power. Power = 7 was selected to ensure a scale-free topology (R² > 0.85). **(C)** Gene dendrogram and WGCNA module assignment. The top panel shows hierarchical clustering of genes. The “Dynamic Tree Cut” color bar indicates initial modules, and “Merged dynamic” shows the final modules after merging similar modules; each color represents one module. **(D)** Heatmap showing the correlation between clinical traits (Control and Diabetic) and identified modules. The turquoise module was most strongly correlated with the diabetic trait. **(E)** Venn diagram displaying the intersection of genes from the turquoise module, PANoptosis-related genes, and DEGs, identifying 13 overlapping genes.

To identify gene modules associated with DKD, we performed weighted gene co-expression network analysis (WGCNA). A total of 63 DKD samples and 53 healthy control samples were included for sample clustering, and outlier samples were removed based on hierarchical clustering (**Figure 3A**). We selected a soft-thresholding power of β = 7 to achieve scale-free topology with R² > 0.85. Using a module eigengene dissimilarity threshold of 0.25, we obtained nine distinct gene modules for further analysis (**Figure 3B**). The cluster dendrogram based on the topological overlap measure (TOM) illustrated the hierarchical relationship between genes and the resulting module structure (**Figure 3C**). Among these, the turquoise module showed the strongest correlation with clinical traits (control vs. DKD) and was chosen as the key module for downstream analysis (**Figure 3D**). By intersecting genes from the turquoise module with PANoptosis-related genes and differentially expressed genes (DEGs), we identified 13 overlapping genes as candidate PANoptosis-related genes (PRGs) (**Figure 3E**).

### 2.4 Screening of Hub DKD-PRGs by machine learning

**Figure 4:**
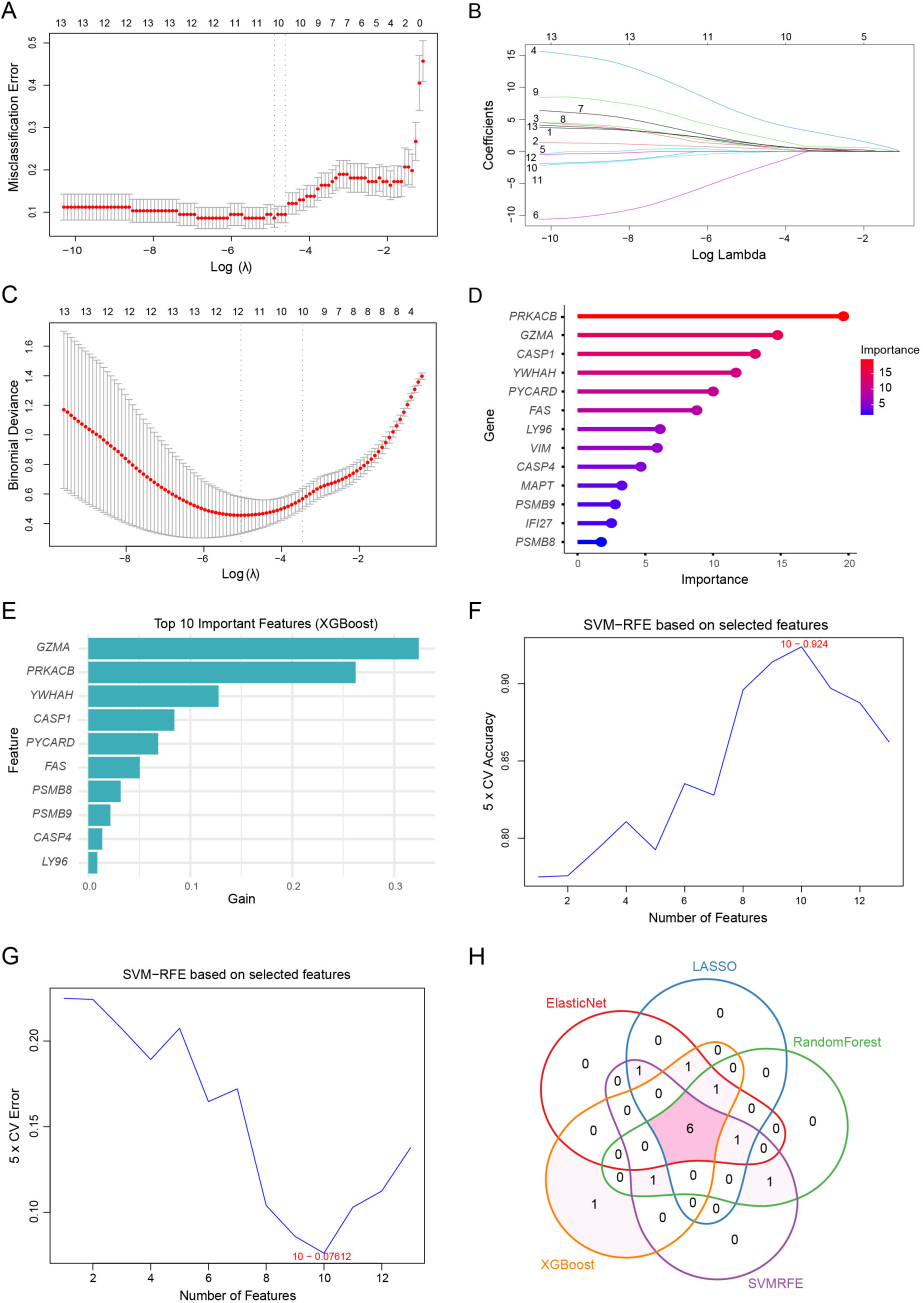
Machine learning–based identification of hub PANoptosis-related genes in DKD. **(A, B)** LASSO regression analysis to identify key feature genes. **(C)** ElasticNet regression analysis with binomial deviance minimization to select optimal variables. **(D)** Feature importance ranking based on the Random Forest algorithm. **(E)** Top 10 important features identified using the XGBoost model. **(F, G)** SVM-RFE algorithm for feature gene selection. **(H)** Venn diagram showing overlapping hub genes identified by five machine learning algorithms (LASSO, ElasticNet, Random Forest, XGBoost, and SVM-RFE), resulting in six consistently selected genes.

We further applied five complementary machine learning algorithms (LASSO, ElasticNet, Random Forest, XGBoost, and SVM-RFE) to prioritize PRGs in DKD. LASSO analysis identified optimal features by minimizing misclassification error and binomial deviance (**Figures 4A, B**), while ElasticNet selected variables based on the balance between L1 and L2 regularization penalties (**Figure 4C**). Random Forest and XGBoost models ranked gene importance based on their contribution to model performance (**Figures 4D, E**), revealing several key genes such as *PRKACB, GZMA*, and *CASP1.* The SVM-RFE method was applied to determine the subset of genes that maximized classification accuracy and minimized error (**Figures 4F, G**), further supporting the robustness of selected features.

By intersecting the top-ranked genes from all five models (**Figure 4H**), we identified a consistent set of six hub DKD-PANoptosis-related differentially expressed genes (DEGs): *YWHAH, PRKACB, PSMB9, FAS, GZMA*, and *CASP1*. These hub genes may serve as promising diagnostic biomarkers and potential therapeutic targets for DKD by capturing key regulatory nodes in PANoptosis-related pathways.

To evaluate their clinical significance, we analyzed Nephroseq v5 datasets. All six genes were significantly upregulated in the tubulointerstitium of DKD patients (**Supplementary Figures 4A, C**). Correlation analyses demonstrated strong negative associations with GFR and positive associations with serum creatinine (**Supplementary Figures 4B, D**), further underscoring their relevance to renal dysfunction and their potential as diagnostic and therapeutic markers in DKD.

### 2.5 Construction and validation of the PANoptosis-related signature risk score for DKD patients

**Figure 5:**
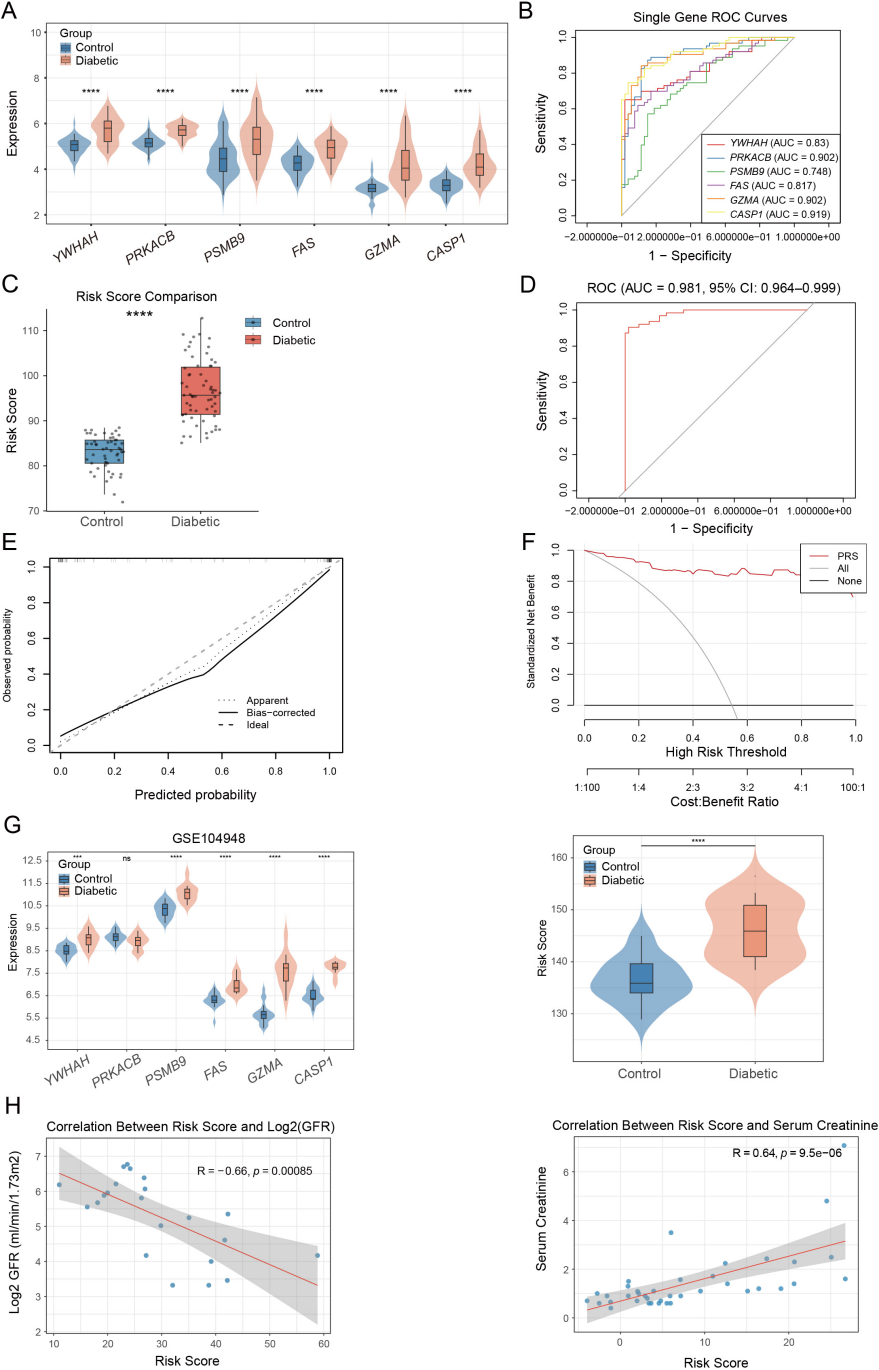
Construction of PANoptosis-related risk score based on core genes. **(A)** Violin plots showing the expression levels of 6 core PANoptosis-related genes (*YWHAH, PRKACB, PSMB9, FAS, GZMA, CASP1*) in control and DKD groups. **(B)** ROC curve analysis of the 6 core PANoptosis-related genes in DKD. **(C)** Boxplot comparing the PANoptosis-related risk score between control and DKD. **(D)** ROC curve analysis of the PRS risk model in DKD. **(E)** Calibration curve of the PRS model. **(F)** Decision curve analysis of the PRS model versus treat-all and treat-none strategies. **(G)** Violin plots showing the expression levels of six hub genes (left) and corresponding risk scores in control and DKD samples (right) from GSE104948. **(H)** Correlation analysis between the PANoptosis-related risk score and renal function, including a negative correlation with glomerular filtration rate (GFR) and a positive correlation with serum creatinine. ***p < 0.001, ****p < 0.0001.

To assess the diagnostic value of hub PANoptosis-related genes in DKD, we evaluated the expression profiles of six core DEGs in normal controls and DKD patients. All six genes were significantly upregulated in the diabetic group (**Figure 5A**), consistent with the findings from the Nephroseq v5 database (**Supplementary Figure 4**). ROC curve analysis further confirmed strong diagnostic efficacy for each gene, with AUC values of *YWHAH* (0.830), *PRKACB* (0.902), *PSMB9* (0.748), *FAS* (0.817), *GZMA* (0.902), and *CASP1* (0.919) (**Figure 5B**). To further enhance diagnostic performance, we constructed a PRS risk score based on the expression levels of the six hub genes. The calculated risk scores were significantly elevated in DKD patients compared to controls (**Figure 5C**). A combined ROC analysis of the integrated risk model yielded an AUC of 0.981 (95% CI: 0.964–0.999), demonstrating excellent diagnostic accuracy (**Figure 5D**). Calibration analysis showed an overall agreement between predicted and observed probabilities, although the model tended to mildly overestimate risk in the mid-to-high probability range; decision curve analysis further indicated a higher net benefit of the PRS model than “treat-all” or “treat-none” strategies across a broad range of threshold probabilities (**Figures 5E, F**).

The robustness of this PRS risk score was further verified in two independent datasets (GSE30529 and GSE104948). In both cohorts, risk scores remained significantly elevated in DKD patients (**Figure 5G**; **Supplementary Figure 5A**), with ROC analyses showing outstanding performance (AUC = 1.000 in GSE30529 and AUC = 0.925 in GSE104948; **Supplementary Figure 5B**). In addition, calibration curves in the validation cohorts showed overall agreement between predicted and observed probabilities (**Supplementary Figure 5B**). Furthermore, we assessed the clinical relevance of the risk score. As shown in **Figures 5H**, the PRS risk score correlated inversely with log_2_(GFR) (R = −0.66, p = 8.5×10^−4^) and positively with serum creatinine (R = 0.64, p = 9.5×10^−6^), indicating that higher PRS values reflect worse renal function.

To evaluate specificity and generalizability beyond healthy controls, we applied the PRS coefficients to a publicly available multi-etiology CKD cohort (GSE99325), which includes hypertensive nephropathy (HT), IgA nephropathy (IgA), rapidly progressive glomerulonephritis (RPGN), membranous glomerulonephritis (MGN), and other etiologies. The PRS robustly discriminated DKD from controls (AUC = 0.972) but showed limited performance for HT and IgA (AUC = 0.412 and 0.469), while yielding higher AUCs in inflammatory glomerular diseases (RPGN AUC = 0.940; MGN AUC = 0.875), suggesting partial overlap with shared injury programs (**Supplementary Figures 5C, E**). To mitigate potential confounding from shared injury programs across CKD etiologies, we performed proxy-adjusted logistic regression using transcriptome-derived fibrosis and inflammation ssGSEA scores as covariates. The PRS remained significantly associated with DKD after adjustment for fibrosis_score (OR = 1.09, 95% CI 1.01–1.17, p = 0.02), inflammation_score (OR = 1.19, 95% CI 1.07–1.32, p < 0.01), or both (OR = 1.16, 95% CI 1.05–1.28, p < 0.01) (**Supplementary Figure 5D**). Collectively, these analyses indicate that although the PRS partially captures shared fibrotic/inflammatory programs, it retains an independent contribution to distinguishing DKD from non-DKD CKD etiologies.

### 2.6 PRS stratification uncovers PANoptosis-linked immune activation in DKD

**Figure 6:**
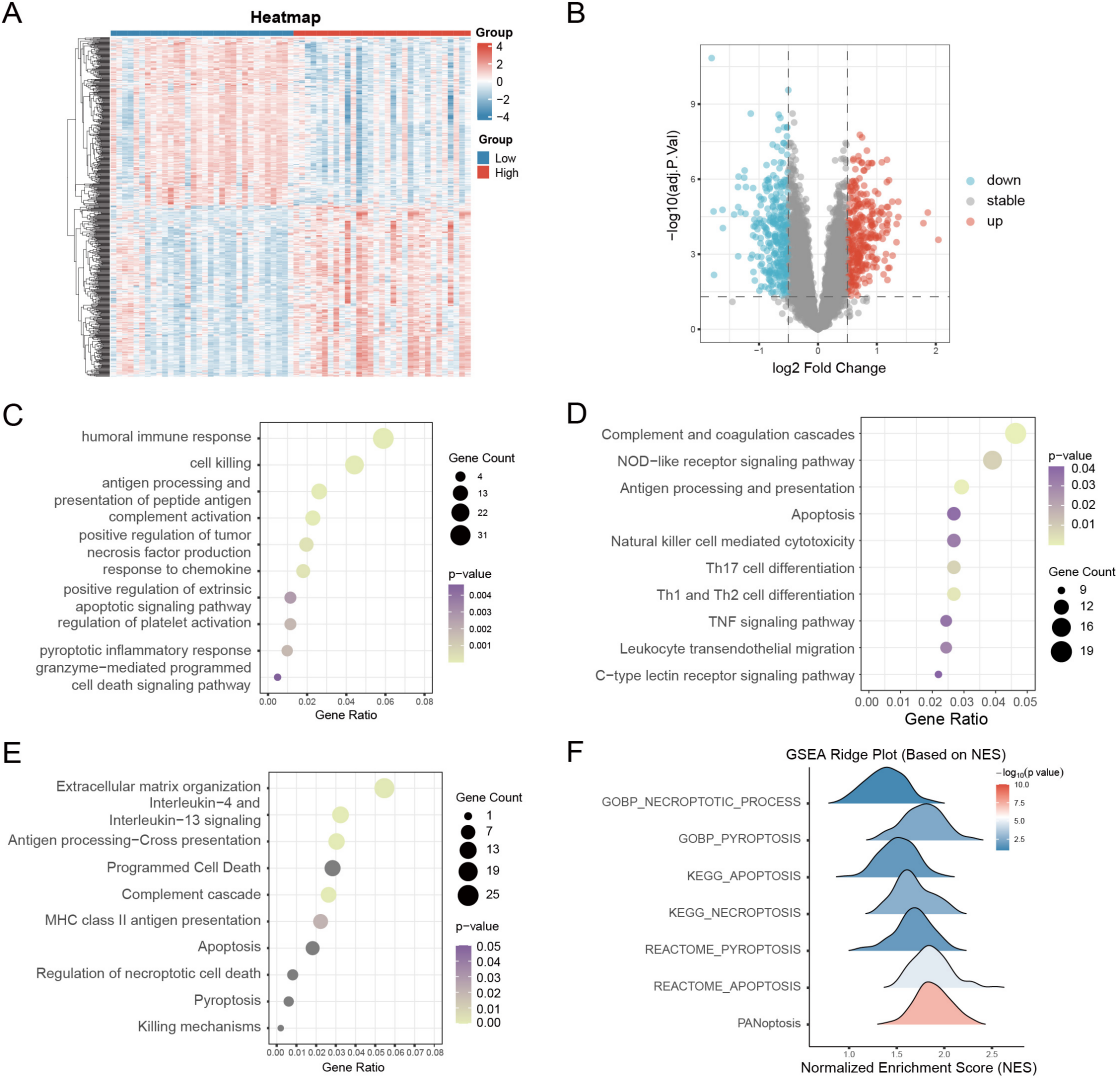
Functional enrichment analysis of DEGs between PANoptosis high- and low-risk groups. **(A, B)** Heatmap and volcano plot of differentially expressed genes (DEGs) between high- and low-risk groups. **(C)** GO enrichment analysis of DEGs, highlighting immune response and cell death–related biological processes. p < 0.05 **(D)** KEGG pathway enrichment analysis of DEGs. p < 0.05 **(E)** Reactome pathway enrichment analysis of DEGs. p < 0.05 **(F)** GSEA ridge plots showing enrichment of PANoptosis-related gene sets across risk groups.

Having established the strong diagnostic performance and clinical relevance of the PRS, we next evaluated whether PRS-defined risk strata capture broader transcriptomic reprogramming and immune perturbations in DKD. According to the median value of the PRS risk score, DKD patients were divided into high- and low-risk groups, yielding 627 DEGs (316 upregulated, 311 downregulated) (**Figures 6A, B**). Enrichment analyses indicated predominant involvement in immune activation and programmed cell death. GO highlighted humoral immunity, complement activation, and pyroptotic response (**Figure 6C**); KEGG analysis revealed prominent immune activation (complement–coagulation, antigen presentation, and NOD/TNF signaling), accompanied by leukocyte migration and NK cytotoxicity (**Figure 6D**). Reactome confirmed apoptosis, pyroptosis, necroptosis, and killing mechanisms (**Figure 6E**). GSEA further showed strong enrichment of pyroptosis, necroptosis, and apoptosis, with PANoptosis most pronounced in high-risk DKD (**Figure 6F**). These findings suggest PANoptosis-driven immune injury underlies transcriptomic reprogramming in DKD.

### 2.7 Landscape of immune dysregulation in DKD

Given the crucial role of immune responses in the development of diabetic kidney disease (DKD) (19), we employed the single-sample Gene Set Enrichment Analysis (ssGSEA) algorithm to quantify the relative enrichment of 28 immune cell types in diabetic and control kidney tissues.

**Figure 7:**
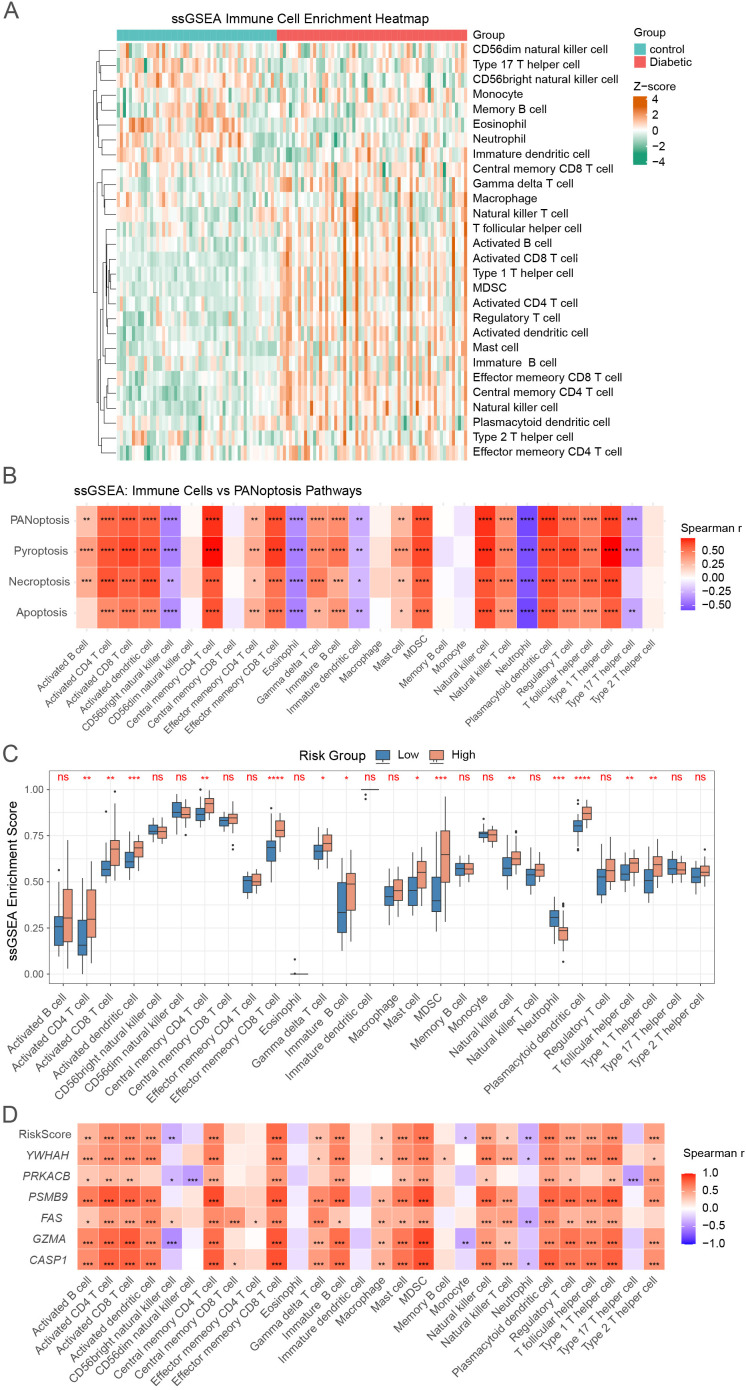
The landscape of immune cell infiltration and PANoptosis associations in DKD. **(A)** Heatmap showing the enrichment scores of 28 immune cell types based on ssGSEA. **(B)** Correlation heatmap between PANoptosis-related pathways and immune cell types. **(C)** ssGSEA analysis of immune cell infiltration scores between high- and low-risk groups. **(D)** Correlation between PANoptosis-related risk score, six hub genes (*YWHAH, PRKACB, PSMB9, FAS, GZMA, CASP1*), and 28 immune cell types. *p < 0.05, **p < 0.01, ***p < 0.001, ****p < 0.0001, ns, not significant.

Heatmap and boxplot analyses revealed marked immune differences, with DKD samples showing increased enrichment of activated CD4^+^/CD8^+^ T cells, γδ T cells, NK cells, and mast cells, indicative of heightened inflammation (**Figure 7A**; **Supplementary Figure 6A**). Correlation analysis demonstrated strong associations between PANoptosis-related pathways (apoptosis, necroptosis, pyroptosis) and proinflammatory immune subsets, suggesting that dysregulated cell death contributes to immune-mediated kidney injury (**Figure 7B**). A correlation matrix further highlighted cooperative interactions among effector T cells, dendritic cells, and cytotoxic populations (**Supplementary Figure 6B**).

Stratification by PRS risk score confirmed these patterns: high-risk patients exhibited significantly greater infiltration of proinflammatory immune cells, and both the PRS score and hub PANoptosis genes positively correlated with immune activation (**Figures 7C, D**). Collectively, these findings indicate that PANoptosis-driven immune dysregulation shapes the inflammatory microenvironment in DKD.

### 2.8 Single-cell transcriptomic landscape of PANoptosis-related hub genes

We then conducted single-cell transcriptomic analysis using publicly available datasets and identified 14 distinct kidney cell populations (**Figure 2D**). The six hub PANoptosis-related genes exhibited striking cell–type–specific expression patterns. *PSMB9, GZMA*, and *CASP1* were predominantly expressed in immune cells, with markedly higher levels in DKD samples compared with controls (**Supplementary Figures 7 C, E, F**). *PRKACB* and *FAS* showed relatively broad expression across multiple cell types (**Supplementary Figures 7B, D**). *YWHAH* displayed stronger expression in DKD than in controls, with enrichment in tubular cells across multiple segments, vascular smooth muscle cells, immune cells, and other tubulointerstitial components (**Supplementary Figure 7A**).

**Figure 8:**
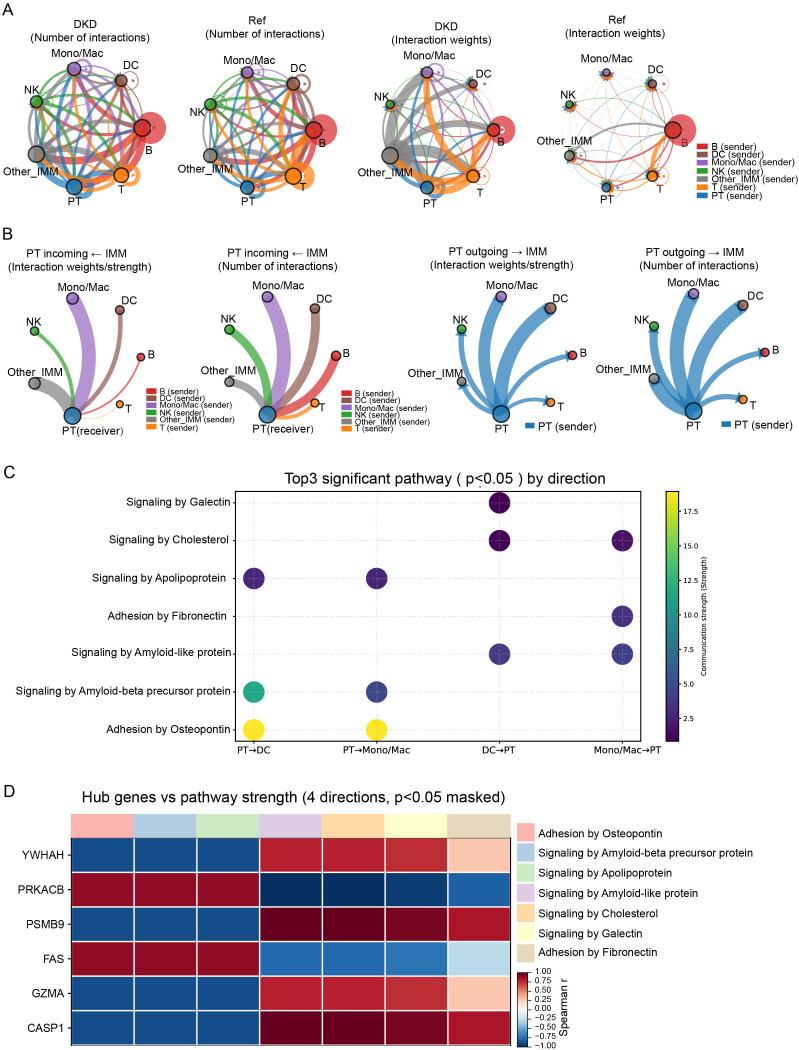
Cell–cell communication between proximal tubule cells and immune populations in DKD. **(A)** Global communication networks. Circle plots summarize inferred intercellular communication among proximal tubule cells (PT) and major immune subsets (Mono/Mac, DC, NK, B, T, and Other_IMM) in DKD and reference/control samples. Networks are shown in two ways: number of interactions (left pair) and interaction weights/strength (right pair). In each plot, node size reflects the total communication level of a cell group, and edge thickness reflects the magnitude of the corresponding interaction (counts or weights/strength), with edge colors indicating the sender cell type. **(B)** PT-centered directional communication. Chord diagrams depict directionality of PT–immune communication, separately showing incoming signals to PT (PT as receiver) and outgoing signals from PT (PT as sender) in DKD and reference/control samples. Edge thickness represents interaction strength (or interaction number, as labeled), allowing comparison of PT→immune and immune→PT communication patterns across conditions. **(C)** Direction-dependent signaling programs. Bubble plot displays the top three significant signaling pathways (p < 0.05) for each communication direction between PT and key immune populations (PT→DC, PT→Mono/Mac, DC→PT, and Mono/Mac→PT). Dot size denotes the contribution/gene count for the pathway, and dot color indicates the communication strength (as shown on the color scale). **(D)** Association between hub genes and communication programs. Heatmap shows the correlation (Spearman’s ρ) between expression of PANoptosis-related hub genes (rows) and the strength of selected signaling pathways (columns) across the four PT–immune directions. Only associations with p < 0.05 are displayed (masked otherwise).

To elucidate intercellular communication and relate hub-gene activity to intercellular signaling, we performed cell–cell communication analysis across proximal tubule cells (PT) and major immune subsets (Mono/Mac, DC, NK, B, T, and Other_IMM) (**Supplementary Figure 3A**). At the global network level, DKD samples showed an overall increase in the number of inferred interactions and interaction weights/strength compared with controls (**Figure 8A**). Notably, communication was evident not only among immune cell subsets (immune–immune) but also as appreciable interactions between tubular cells and immune populations. Focusing on PT-centered communication, directional analysis indicated that both PT-to-immune outgoing signals and immune-to-PT incoming signals were bidirectionally active in DKD; among these, PT–Mono/Mac and PT–DC interactions were particularly prominent in terms of interaction number and/or strength (**Figure 8B**).

We next interrogated signaling programs potentially underlying PT communication with Mono/Mac and DC. Several pathways showed direction-dependent patterns, with Signaling by Apolipoprotein, Adhesion by Osteopontin, and Signaling by Amyloid-beta precursor protein primarily observed in the PT→Mono/Mac and PT→DC directions (**Figure 8C**). In addition, signaling by Galectin and ECM-adhesion modules (including Adhesion by Osteopontin and Adhesion by Fibronectin) recurred across multiple communication directions, indicating that ECM-adhesion- and metabolism/lectin-related signals represent prominent features of immune–tubular communication in DKD. Finally, we assessed the relationship between hub-gene expression and pathway communication strength and observed distinct, direction-dependent association patterns across PT–immune directions (**Figure 8D**), linking PANoptosis-related hub genes with the broader immune–tubular signaling network.

### 2.9 Validation of hub PANoptosis-related genes in DKD mouse models

**Figure 9:**
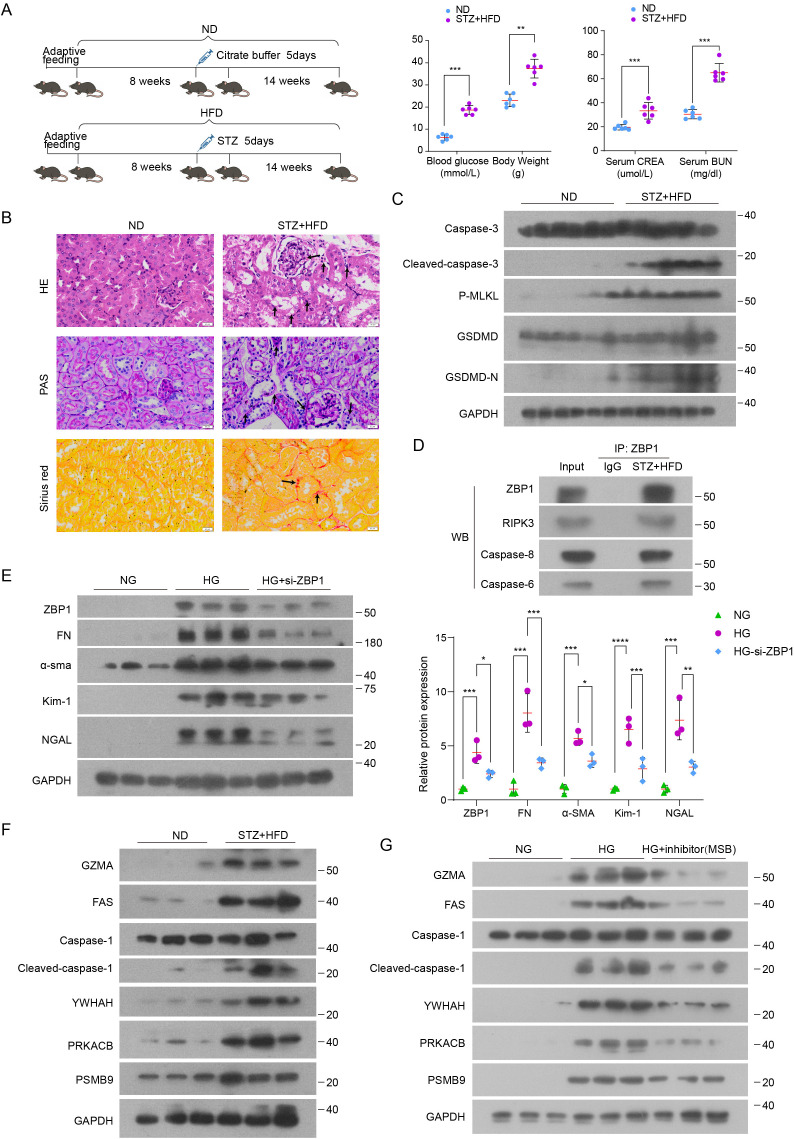
Validation of Hub PANoptosis-Related Genes in DKD Mouse Models. **(A)** Left, schematic illustration of the STZ+HFD-induced DKD model. Right, blood glucose, body weight, serum creatinine (CREA), and blood urea nitrogen (BUN) in the indicated mouse groups (ND vs STZ+HFD). **(B)** Representative kidney sections stained with hematoxylin and eosin (H&E), periodic acid–Schiff (PAS), and Sirius red. **(C)** Representative immunoblots of PANoptosis-associated execution markers in kidneys from ND and STZ+HFD mice, including apoptosis (caspase-3/cleaved caspase-3), necroptosis (p-MLKL), and pyroptosis (GSDMD/GSDMD-N). **(D)** Co-immunoprecipitation (co-IP) using ZBP1 as bait from STZ+HFD kidney lysates (with IgG as control), followed by immunoblotting for RIPK3, caspase-8, and caspase-6, indicating a ZBP1-centered PANoptosome-like complex *in vivo*. **(E)** Left, immunoblotting of ZBP1 and fibrosis/injury markers (FN, α-SMA, KIM-1, and NGAL) in HK-2 cells cultured under normal glucose (NG), high glucose (HG), or HG with si-ZBP1. Right, quantification of protein levels. **(F)** Immunoblotting of hub PANoptosis-related proteins (GZMA, FAS, CASP1/cleaved CASP1, YWHAH, PRKACB, and PSMB9) in kidneys from ND and STZ+HFD mice. **(G)** Immunoblotting of hub PANoptosis-related proteins in HK-2 cells cultured under NG, HG, or HG plus MSB (PANoptosis inhibitor). P values are indicated as: *P < 0.05; **P < 0.01; ***P < 0.001; ****P < 0.0001.

To validate hub PANoptosis-related genes at the experimental level, we established a diabetic kidney disease (DKD) model using streptozotocin combined with a high-fat diet (STZ+HFD) (**Figure 9A**). Compared with normal diet (ND) controls, STZ+HFD mice showed hyperglycemia and increased body weight, accompanied by elevated serum creatinine and blood urea nitrogen and increased urinary albumin-to-creatinine ratio (UACR) (**Figure 9A**; **Supplementary Figure 8A**), confirming metabolic disturbance and renal dysfunction. Histological analyses further demonstrated overt renal injury and enhanced fibrotic remodeling in DKD kidneys, with significantly higher tubular injury scores and fibrosis indices than controls (**Figure 9B**; **Supplementary Figure 8B**).

To further determine whether PANoptosis-associated signaling is activated at the protein level in diabetic kidneys, we assessed representative molecular markers of pyroptosis (GSDMD cleavage), apoptosis (caspase-3 cleavage), and necroptosis (MLKL phosphorylation [p-MLKL]). Immunoblotting showed increased cleaved GSDMD, cleaved caspase-3, and p-MLKL in STZ+HFD kidneys (**Figure 9C**; **Supplementary Figure 8C**). To provide more direct evidence for a PANoptosome-like execution complex in DKD kidneys, we performed co-immunoprecipitation (co-IP) using ZBP1 as the bait in STZ+HFD renal tissues. ZBP1 co-precipitated with RIPK3 and caspase-8, along with caspase-6 (**Figure 9D**), supporting the formation of a ZBP1-centered protein complex associated with PANoptosis execution *in vivo*.

To explore the functional relevance of PANoptosis signaling in renal tubular epithelial cells, we performed *in vitro* experiments in HK-2 cells exposed to high glucose (HG). HG markedly induced the expression of Z-DNA-binding protein 1 (ZBP1), a key PANoptosome component, and increased fibrotic markers (FN, α-SMA) and injury markers (Kim-1, NGAL), whereas siRNA-mediated ZBP1 silencing attenuated these HG-induced changes (**Figure 9E**). In addition, to pharmacologically interrogate PANoptosis, we treated HG-stimulated tubular cells with the PANoptosis inhibitor MSB (20). MSB markedly reduced the HG-induced upregulation of the hub-gene proteins (GZMA, FAS, CASP1, YWHAH, PRKACB, and PSMB9) (**Figure 9G**; **Supplementary Figure 8G**), providing inhibitor-based evidence that these hub genes are functionally linked to PANoptosis activation in tubular injury.

Finally, at the molecular level, RT-qPCR analysis showed robust upregulation of six hub PANoptosis-related genes in the STZ + HFD group (**Supplementary Figure 8D**). Consistently, these genes were also significantly upregulated in db/db mice compared with db/m controls (**Supplementary Figure 8E**). In parallel, immunoblotting confirmed increased protein expression of these hub genes in DKD kidneys (**Figure 9F**; **Supplementary Figure 8F**).

### 2.10 Regulatory networks and druggability of panoptosis hub genes

Finally, we delineated the regulatory landscape and therapeutic tractability of the panoptosis hub genes. We first queried ENCORI (starBase) for miRNA–mRNA interactions and found that, among all hub genes, only *FAS, YWHAH*, and *PRKACB* were supported by experimental miRNA–target evidence (**Supplementary Table 4**). Based on these interactions, we constructed an miRNA–mRNA regulatory network and identified nine miRNAs that may coordinately regulate *FAS, YWHAH*, and *PRKACB* (**Supplementary Figure 10A**). In parallel, transcription factors potentially regulating each hub gene were retrieved from hTFtarget and integrated in Cytoscape to generate a TF–hub gene regulatory network (**Supplementary Figure 10B**, **Supplementary Table 5**).

We then evaluated the druggability of the panoptosis hub genes using DrugnomeAI. Across generic models trained on different druggable gene sets (Tclin, Tchem, Tier 1–3A, and their combinations), *CASP1, FAS, PSMB9*, and *PRKACB* consistently achieved high percentile scores and were classified as druggable targets (**Supplementary Figure 10C**). Modality-specific models indicated that *CASP1* and *FAS* are amenable to both antibody-based and small-molecule therapies, whereas *PSMB9* and *PRKACB* are primarily suited for small-molecule development (**Supplementary Figure 10D**). Guided by these druggability predictions, we next queried DGIdb to retrieve drug–gene interaction records for the panoptosis hub genes and compiled a list of potentially actionable compounds (**Supplementary Table 6**). Applying our predefined prioritization scheme (Methods 3.12), we highlighted three Tier 1 candidates: aspirin and atorvastatin calcium trihydrate for FAS, and diacetylrhein for CASP1 (**Supplementary Table 6**). Collectively, these results provide a preliminary pharmacopeia centered on panoptosis hub genes for future experimental validation and drug-repurposing efforts, with the understanding that DGIdb-based links reflect curated interaction evidence and require context-specific validation (e.g., renal accessibility, safety, and mechanistic plausibility).

## 3 Materials and methods

### 3.1 Data acquisition

Microarray-based mRNA expression profiles from the tubulointerstitial compartment of human kidney tissues were obtained from four GEO datasets (GSE30122, GSE104954, GSE99325, and GSE47185) in the Gene Expression Omnibus (GEO) database (http://www.ncbi.nlm.nih.gov/geo/). For validation, one tubulointerstitial dataset (GSE30529) and one glomerular dataset (GSE104948) were additionally included, together with single-cell transcriptomic data of DKD and healthy controls retrieved from public databases (18). Detailed dataset information is summarized in **Supplementary Table 1**.

PANoptosis-related genes were curated from KEGG, GO BP, Reactome, and published reviews (21), including GOBP_NECROPTOTIC_PROCESS (GO:0070266), GOBP_PYROPTOSIS (GO:0070269), REACTOME_PYROPTOSIS (R-HSA-5620971), REACTOME_APOPTOSIS (R-HSA-109581), KEGG_Apoptosis (hsa04210) and KEGG_Necroptosis (hsa04217). These gene sets were retrieved via MSigDB (v2024.1. Hs, accessed on May 30, 2025), corresponding to the GO/KEGG/Reactome terms listed above. After integration and removal of duplicate entries across gene sets, 416 unique genes were retained, encompassing genes annotated to apoptosis (234), necroptosis (186), and pyroptosis (57). The complete gene list is available in **Supplementary Table 2**. Clinical data for DKD patients were further retrieved from the Nephroseq v5 online database.

### 3.2 Data processing

For microarray mRNA expression data, batch effects were removed using the SVA package (22). Differentially expressed genes (DEGs) were then identified with the R package “limma”, and visualized using “ggplot” to generate volcano plots. Genes with adjusted P < 0.05 and |log2FC| > 0.5 were considered statistically significant. Importantly, all feature selection steps were performed using the combined training datasets only (GSE104954, GSE30122, GSE47185, and GSE99325), and the validation cohorts were not involved in any parameter estimation, feature selection, or model tuning. as illustrated in **Supplementary Material 8**.

We utilized the archived version of the Kidney Cell Atlas available in Cellxgene. To focus specifically on diabetic kidney disease (DKD), we first excluded samples from other kidney conditions, retaining only cells from DKD patients and healthy controls. To quantify the three major components of PANoptosis—pyroptosis, apoptosis, and necroptosis—we applied the scoring approach implemented in *scanpy.tl.score_genes*. Briefly, for each curated gene set, the score was defined as the average expression of the signature genes minus the average expression of a corresponding reference set of genes. Individual component scores (pyroptosis, apoptosis, necroptosis) were calculated using their respective curated signatures, while the PANoptosis score was obtained from the combined signature of all three processes. To illustrate potential crosstalk among the three components at the single-cell level—where a single cell may simultaneously exhibit features of more than one death pathway—we generated pairwise composite visualizations of the scores using divergent colormaps. In these plots, cells with high values for only one of the two scores appeared primarily in red or green, whereas cells with high values for both scores were represented by blended colors, indicating co-activation. Cell–cell communication analysis was performed using the omicverse CellPhoneDB/CellChat-style visualization module (omicverse.pl._cpdbviz/CellChatViz).

### 3.3 Pathway and functional enrichment analysis

We utilized the R package “clusterProfiler” to conduct Gene Ontology (GO), Kyoto Encyclopedia of Genes and Genomes (KEGG), and Reactome pathway enrichment analyses. Additionally, we performed gene set enrichment analysis (GSEA) to identify the underlying pathways, with the threshold for significant terms being adjusted p-value <0.05.

### 3.4 Construction of the co-expression network and key modules identification by WGCNA

To identify gene modules associated with DKD, we performed WGCNA using the R package “WGCNA” (23). Outlier samples and low-quality genes were removed using the goodSamplesGenes function. Sample clustering was conducted to detect potential outliers, and a soft-thresholding power was selected using the pickSoftThreshold function to ensure scale-free topology (R² > 0.85). Gene modules were identified using the blockwiseModules function with the following key parameters: soft-thresholding power = 7, minimum module size = 50, and merge cut height = 0.25. Module eigengenes were calculated, and their correlations with clinical traits (control vs. DKD) were assessed to determine phenotype-associated modules.

### 3.5 Machine learning–based identification of hub genes

We applied five machine learning algorithms: LASSO, Elastic Net, Random Forest, XGBoost, and SVM-RFE. LASSO and Elastic Net were conducted with 10-fold cross-validation, and genes with non-zero coefficients at λ_min were retained. Random Forest (ntree = 500) and XGBoost were used to rank features by importance, and SVM-RFE was used to identify optimal gene subsets by recursive feature elimination. The final hub genes were defined as the intersection of genes prioritized by all five methods.

### 3.6 Analysis of immune cell infiltration

Immune cell infiltration was quantified using single-sample Gene Set Enrichment Analysis (ssGSEA), which estimates the relative enrichment of immune cell signatures in each sample. Marker gene sets for 28 immune cell types were obtained from previously published studies (23). ssGSEA enrichment scores were computed from the gene expression matrix using the R package GSVA, allowing comparison of immune infiltration patterns between groups. Between-group differences in ssGSEA scores were assessed using the two-sided Wilcoxon rank-sum test, with Benjamini–Hochberg false discovery rate (FDR) correction applied across immune cell types. Correlations were evaluated using Spearman’s rank correlation.

### 3.7 Construction of the PRS risk score model

The PRS risk score was developed using multivariable logistic regression (24). The score was defined as a linear combination of the expression levels of the selected genes and their corresponding multivariable Logistic coefficients:


$$Risk\;Score\;=\sum_{i=1}^{n}\beta_{i}\times Exp_{i}$$

Based on this approach, the final model incorporated six hub PANoptosis-related genes, and the risk score was calculated as: PRS  Risk Score=(4.4312×YWHAH)+(8.6692×PRKACB)−(4.6435×PSMB9)+(2.1184×FAS)+(1.1198×GZMA)+(7.4013×CASP1).Proxy-adjusted logistic regression analyses. To account for potential confounding by shared injury programs across CKD etiologies, we performed proxy-adjusted logistic regression analyses using transcriptome-derived fibrosis and inflammation scores as covariates. Fibrosis_score and inflammation_score were computed from bulk transcriptomes by single-sample gene set enrichment analysis (ssGSEA) using the GSVA framework (method = “ssgsea”). As previously described (25), the fibrosis score was calculated based on an extracellular matrix (ECM) gene set assembled from the NABA ECM categories, including NABA_COLLAGENS, NABA_ECM_GLYCOPROTEINS, and NABA_PROTEOGLYCANS (merged and de-duplicated). The inflammation score was calculated using the HALLMARK_INFLAMMATORY_RESPONSE gene set. We fitted logistic regression models for DKD status (DKD vs non-DKD CKD) including: Model 1 (DKD ~ PRS), Model 2 (DKD ~ PRS + fibrosis_score), Model 3 (DKD ~ PRS + inflammation_score), and Model 4 (DKD ~ PRS + fibrosis_score + inflammation_score). Odds ratios (ORs) and 95% confidence intervals for the PRS term were reported.

### 3.8 Construction of receiver operating characteristic curve

To evaluate the diagnostic performance of individual hub genes, ROC curve analysis and AUC (area under the curve) calculation were performed using the “pROC” package in R. For each gene, a univariate ROC curve was plotted, and corresponding AUC values were calculated.

### 3.9 DKD mouse model

Male mice were randomly assigned to two experimental groups. The control group (CTL + Veh + ND, n = 6) was maintained on a standard chow diet, while the DKD group (CTL + STZ + HFD, n = 6) received a high-fat diet (Research Diets, Guangdong Medical Animal Center, Guangzhou, China) for 22 weeks. After eight weeks of HFD feeding, DKD mice were administered intraperitoneal injections of STZ (40 mg/kg; Sigma-Aldrich, St. Louis, MO, USA) dissolved in freshly prepared ice-cold sodium citrate buffer (pH 4.5) once daily for five consecutive days. Control animals received equivalent volumes of sodium citrate buffer alone. In parallel, twelve-week-old male db/db mice were obtained from Cyagen Company (Guangzhou, China), with age-matched db/m mice serving as controls, to another model of type 2 diabetes.

### 3.10 Histopathological staining

Following sacrifice, mouse kidneys were immediately immersed in 4% paraformaldehyde, dehydrated, and embedded in paraffin. Paraffin blocks were sectioned at a thickness of 4 μm for subsequent histological staining. For H&E staining, hematoxylin and eosin were applied for 20 minutes and 45 seconds, respectively, following standard procedures. PAS staining was carried out using a commercial kit (Solarbio, G1281), and collagen deposition was assessed by Sirius red staining (BASO, BA4356) according to the manufacturer’s instructions (26).

### 3.11 RNA extraction and quantitative real-time PCR

Total RNA was isolated from mouse kidney tissues using TRIzol reagent (Vazyme, Nanjing, China) followed by chloroform extraction. RNA was precipitated with isopropanol, washed, and resuspended in nuclease-free double-distilled water (ddH₂O). Complementary DNA (cDNA) was synthesized from 1 μg of total RNA using a reverse transcription kit (Vazyme, Nanjing, China). The resulting cDNA was diluted 1:10 and subjected to quantitative real-time PCR using SYBR Green Master Mix (Vazyme, Nanjing, China) on a Roche Applied Science LightCycler 480 Fast Real-Time PCR System. GAPDH served as the internal control, and the primer sequences used are listed in **Supplementary Table 3**.

### 3.12 Prediction of miRNA/TF regulatory networks and druggability of PANoptosis hub genes

miRNA–hub gene interactions were predicted using ENCORI database (https://rnasysu.com/encori/). For each panoptosis hub gene, we used the miRNA–target Quick Search module with the following settings: species restricted to mammal/human (hg38), miRNA = all, CLIP-Data ≥ 1, Degradome-Data ≥ 0, pan-Cancer ≥ 0, and programNum ≥ 1. miRNA–mRNA Networks were visualized by using the Cytoscape (v 3.10.2) software. Transcription factors (TFs) potentially regulating the hub genes were obtained from the hTFtarget (databasehttps://guolab.wchscu.cn/hTFtarget/#!/target). TF networks were visualized by using the Cytoscape (v 3.10.2) software.

Finally, the druggability of individual PANoptosis hub genes was assessed using DrugnomeAI, a machine learning–based framework for druggability prediction (27). Gene-level druggability scores and therapeutic modality predictions were retrieved from DrugnomeAI. To link hub genes with candidate therapeutic agents in DKD, drug–gene interaction information was extracted from the Drug–Gene Interaction Database (DGIdb; https://www.dgidb.org/), and potential drug–hub gene pairs were systematically compiled. Drug prioritization strategy: Candidate drug–target interactions (DTIs) were prioritized into three tiers. Tier 1 included approved drugs with diabetes- and/or diabetes complication-related indications. Tier 2 included approved drugs without diabetes-related indications but with interaction_score ≥ the 75th percentile among approved records. Tier 3 comprised all remaining DTIs, including non-approved drugs and approved drugs failing the Tier 1/2 criteria.

### 3.13 Immunoblotting

Proteins were extracted from cells (or kidney tissues) using RIPA lysis buffer supplemented with protease and phosphatase inhibitors. Protein concentration was measured by a BCA assay. Equivalent amounts of protein were denatured in loading buffer, separated by SDS–PAGE, and transferred onto nitrocellulose membranes. After blocking, membranes were incubated with primary antibodies at 4 °C overnight (anti-NGAL, Abcam; anti-caspase-1, Abmart; anti-GSDMD, Abcam; anti-GAPDH, Proteintech; anti-ZBP1, Proteintech; anti-fibronectin/FN, Proteintech; anti-α-SMA, Proteintech; anti-Kim-1, Boster; anti-YWHAH, Solarbio; anti-PRKACB, Solarbio; anti-PSMB9, Solarbio; anti-GZMA, Proteintech; anti-caspase-3, Cell Signaling Technology; anti-FAS, Abmart; anti-MLKL, abcam), followed by appropriate HRP-conjugated secondary antibodies. Signals were developed using chemiluminescence and quantified by ImageJ.

### 3.14 Cell culture and treatment

HK-2 cells were maintained in DMEM/F-12 supplemented with 10% FBS and 1% penicillin–streptomycin at 37 °C with 5% CO₂. For glucose stimulation, cells were cultured in normal glucose (NG; 5.6 mmol/L D-glucose) or high glucose (HG; 30 mmol/L D-glucose) medium for the indicated time periods. For knockdown experiments, cells were transfected with siRNA using Lipo8000 (Beyotime) according to the manufacturer’s protocol and then subjected to NG or HG treatment as indicated.

### 3.15 Statistical analysis

All data are presented as mean ± SD. Graphs were generated using GraphPad Prism 9 (GraphPad Software, CA, USA), while statistical analyses were conducted with SPSS 23.0 (SPSS Inc., Chicago, USA). Differences between the two groups were evaluated using unpaired Student’s *t*-tests, and *p* < 0.05 was considered statistically significant.

## 4 Discussion

The pathogenesis of diabetic kidney disease (DKD) is multifactorial, with increasing evidence that renal tubular injury precedes glomerular damage and microalbuminuria (28, 29). Multiple programmed cell death (PCD) pathways—including apoptosis, pyroptosis, and necroptosis—are involved in tubular and interstitial injury, but most studies have examined them in isolation (30–33). PANoptosis, a recently defined inflammatory PCD integrating these three pathways via the PANoptosome, exemplifies their extensive molecular crosstalk (15). Although PANoptosis has been linked to various kidney diseases and shown to drive podocyte injury in DKD, its role in tubular and tubulointerstitial injury under diabetic conditions remains unclear (16). Here, we systematically evaluate the crosstalk among the three major cell death pathways in DKD, defining PANoptosis features in renal tubules and interstitium to provide new mechanistic insight.

In the study, we identified differentially expressed genes (DEGs) in DKD renal tubules and tubulointerstitium, revealing robust activation of apoptosis-, pyroptosis-, and necroptosis-associated pathways. Approximately 13% of related genes were shared by at least two pathways, and seven core genes were common to all three, indicating substantial molecular overlap. Consistent with our observations, previous studies have demonstrated potential mechanisms of crosstalk among these pathways (34–36). For instance, caspase-8 serves as a central regulatory hub that not only initiates apoptosis through caspase-3 activation but also suppresses necroptosis by cleaving RIPK1 and RIPK3 (37, 38). Under specific inflammatory conditions, caspase-8 may also interact with inflammatory caspases to modulate pyroptosis (39, 40). Single-cell RNA sequencing further demonstrated that individual renal cells frequently exhibit concurrent activation of multiple PCD pathways, with PANoptosis showing the most extensive enrichment across cell populations. This redundancy suggests that inhibition of a single PCD pathway may be insufficient, as alternative death programs can compensate to ensure cell fate execution (41, 42). However, because apoptosis, pyroptosis, and necroptosis signatures are concurrently enriched, our current transcriptomic data alone cannot determine which modality contributes most to DKD injury. This will require execution-level studies that selectively perturb each arm and quantify pathway-specific protein readouts together with injury phenotypes under defined diabetic conditions. Collectively, these findings position PANoptosis as a potential integrative driver of DKD-associated tubular injury.

Building on this integrated landscape, we next sought to identify PANoptosis-related biomarkers for clinical stratification. Through an integrative approach combining WGCNA and multiple machine learning algorithms, we identified six hub genes: *YWHAH, PRKACB, PSMB9, FAS, GZMA*, and *CASP1*. These genes were strongly associated with renal dysfunction, as evidenced by their negative correlation with GFR and positive correlation with serum creatinine. ROC analyses confirmed their high diagnostic accuracy. To improve clinical utility, we developed a composite diagnostic score (PRS Risk Score) based on the six genes, which demonstrated excellent performance in both the training and validation cohorts. Notably, although PSMB9 was upregulated in DKD, it received a negative weight within the multigene score. This pattern is consistent with coefficient redistribution under strong inter-gene correlation, where the score reflects the conditional contribution of each gene rather than its marginal direction alone. In support of this interpretation, PSMB9 showed a positive association when considered alone (β = 1.127) but became negative in the 6-gene score (β = −4.643), and it was highly correlated with other PRS genes (**Supplementary Figures 5D, E**). Our results showed that higher scores were associated with increased immune infiltration and impaired renal function. Single-cell RNA sequencing further revealed cell-type-specific expression of these genes, particularly within immune cells, renal tubular cells, and tubulointerstitial cells. Their upregulation was validated in both STZ-induced and db/db diabetic mouse models, lending additional *in vivo* support to their pathophysiological relevance.

We also noted very high AUCs in some cohorts, which may indicate optimistic performance. We therefore reported 95% confidence intervals and complemented discrimination with calibration and decision-curve analyses; nevertheless, such high AUCs may partly reflect strong transcriptomic case–control separation, limited sample size, and cohort-specific effects. In the multi-etiology CKD cohort, relatively high AUCs in RPGN/MGN suggest that the PRS may partially capture a shared inflammatory/fibrotic injury axis, which is biologically plausible given that PANoptosis-related immune-inflammatory programs can be activated across kidney disease etiologies (43). Using ssGSEA-derived fibrosis and inflammation scores as proxy covariates, the PRS remained significantly associated with DKD after proxy adjustment, supporting an independent contribution beyond these shared injury programs. Overall, the PRS is best interpreted as a transcriptome-based tool for DKD discrimination/stratification within CKD backgrounds, while acknowledging partial cross-etiology signals and the need for validation in larger, clinically harmonized cohorts.

Among the hub genes, *YWHAH* encodes the 14-3-3η protein, an adaptor regulating signal transduction, cell cycle, and cell death (44). Functionally, *YWHAH* enhances type I interferon signaling via MDA5, which may promote PANoptosome formation through IRF1-ZBP1 activation (45). *PRKACB* encodes the catalytic subunit β of protein kinase A (PKA) (46). By phosphorylating p65 at Ser276, it enhances NF-κB transcriptional activity, promoting inflammation in chronic diseases (47). *PSMB9* encodes the β1i subunit of the immunoproteasome, which is upregulated under inflammatory conditions (48, 49). While its direct role in PANoptosis is unclear, IFN-γ—a known PANoptosis inducer—can promote the switch from constitutive proteasomes to immunoproteasomes (*PSMB8/9/10*), suggesting that *PSMB9* may contribute to IFN-γ–driven PANoptosis (50).

*GZMA, FAS*, and *CASP1* are key regulators of distinct programmed cell death (PCD) pathways with potential convergence on PANoptosis (51–53). *GZMA*, a serine protease secreted by cytotoxic T and NK cells, induces caspase-independent apoptosis via SET complex disruption, triggers pyroptosis through *GSDMB* cleavage (54). *FAS*, a canonical death receptor in extrinsic apoptosis, can initiate necroptosis through TRAIL–FADD–Caspase-8 signaling when Caspase-8 is inhibited; notably, FADD is also a PANoptosome component (41, 55). *CASP1*, the central pyroptosis effector, which can also drive apoptosis via caspase-7 activation and participates in multiple PANoptosome assemblies (ZBP1-, RIP1-, and NLRP12-dependent) in response to diverse inflammatory stimuli (39, 56).

Increasing evidence links dysregulated immune responses to DKD pathogenesis and progression (57). As a highly inflammatory form of programmed cell death, PANoptosis amplifies immune signaling through cytokine release and inflammasome activation, promoting immune cell recruitment and sustained inflammation (55, 58). We therefore hypothesize that PANoptosis in DKD may not only mediate intrinsic cell death but also shape the renal immune microenvironment. In our analysis, DEGs from DKD kidneys showed marked enrichment of proinflammatory pathways, and immune infiltration profiling revealed elevated proinflammatory cell populations compared with controls. Interestingly, PANoptosis and its associated cell death pathways exhibited similar immune signatures, further supporting the notion of functional crosstalk among these death mechanisms. Correlation analysis further demonstrated that higher PANoptosis-related risk scores were associated with increased infiltration of proinflammatory immune cells, underscoring both mechanistic relevance and diagnostic potential.

Despite the strengths of our integrative analysis, several limitations should be noted. First, although we initially explored the crosstalk of PANoptosis-related death pathways in DKD and identified key hub genes, their specific mechanisms remain unclear. Future studies should validate causality by perturbing individual hubs (siRNA/CRISPR knockdown or overexpression) in diabetic tubular models *in vitro* and *in vivo*. Second, while we have added canonical protein readouts of pyroptosis/apoptosis/necroptosis, transcriptomic enrichment alone cannot demonstrate PANoptosome assembly or pathway execution, because PANoptosis is defined by protein-complex formation and post-translational activation. Direct assessment of PANoptosome formation will be important in future work. Finally, our druggability and drug–gene interaction analyses are computational and hypothesis-generating, and therefore do not establish efficacy or mechanistic effects on PANoptosis in DKD. Future work should prioritize clinically actionable candidates and test them in diabetic tubular models *in vitro* and *in vivo*, with PANoptosis-associated protein readouts and renal injury phenotypes.

## 5 Conclusion

In summary, we systematically mapped the perturbations and crosstalk among PANoptosis-related cell death pathways in renal tubular and tubulointerstitial compartments in DKD, and identified six hub genes (*GZMA, FAS, CASP1, YWHAH, PRKACB*, and *PSMB9*). We established a PANoptosis-related risk score (PRS) that closely tracks renal dysfunction and immune activation, and confirmed the spatial distribution and experimental relevance of these genes in diabetic mouse kidneys, supporting their potential as robust tissue biomarkers. Integrative regulatory and druggability analyses using DrugnomeAI further pinpoint *CASP1, FAS, PSMB9*, and *PRKACB* as central, experimentally tractable and pharmacologically actionable PANoptosis nodes. Together, these findings link aberrant panoptosis to immune–fibrotic remodeling in DKD and nominate panoptosis hub genes as promising biomarkers and therapeutic entry points for future translational studies.

## Data Availability

The datasets presented in this study can be found in online repositories. The names of the repository/repositories and accession number(s) can be found in the article/**Supplementary Material**.
