# Supplementary material for: Identification of PANoptosis hub genes driving immune activation and tubulointerstitial injury in diabetic kidney disease by integrative bioinformatics and machine learning
Source: Front Immunol. 2026 Mar 9;17:1759781. doi: 10.3389/fimmu.2026.1759781 (PMC13006297; doi:10.3389/fimmu.2026.1759781)
Supplement: Supplementary file 2 [file Table1.docx]

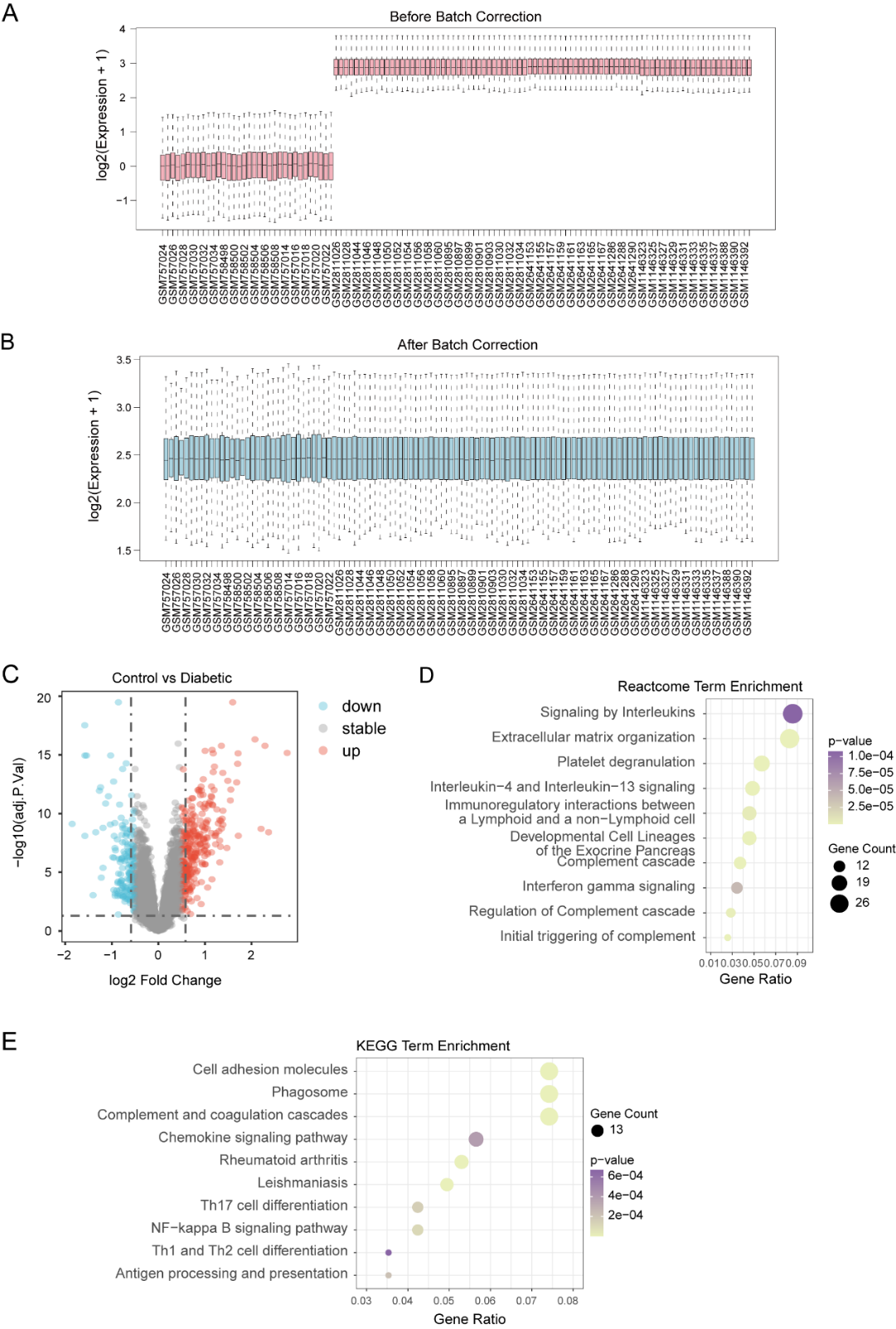


**Supplementary Figure 1** (A, B) Boxplot distributions of gene expression values before and after batch effect correction across four GEO datasets. (C) Volcano plot of DEGs between the control and DKD groups. (D, E) Reactome and KEGG enrichment analyses of DEGs, highlighting immune activation and cell death–related signaling pathways.


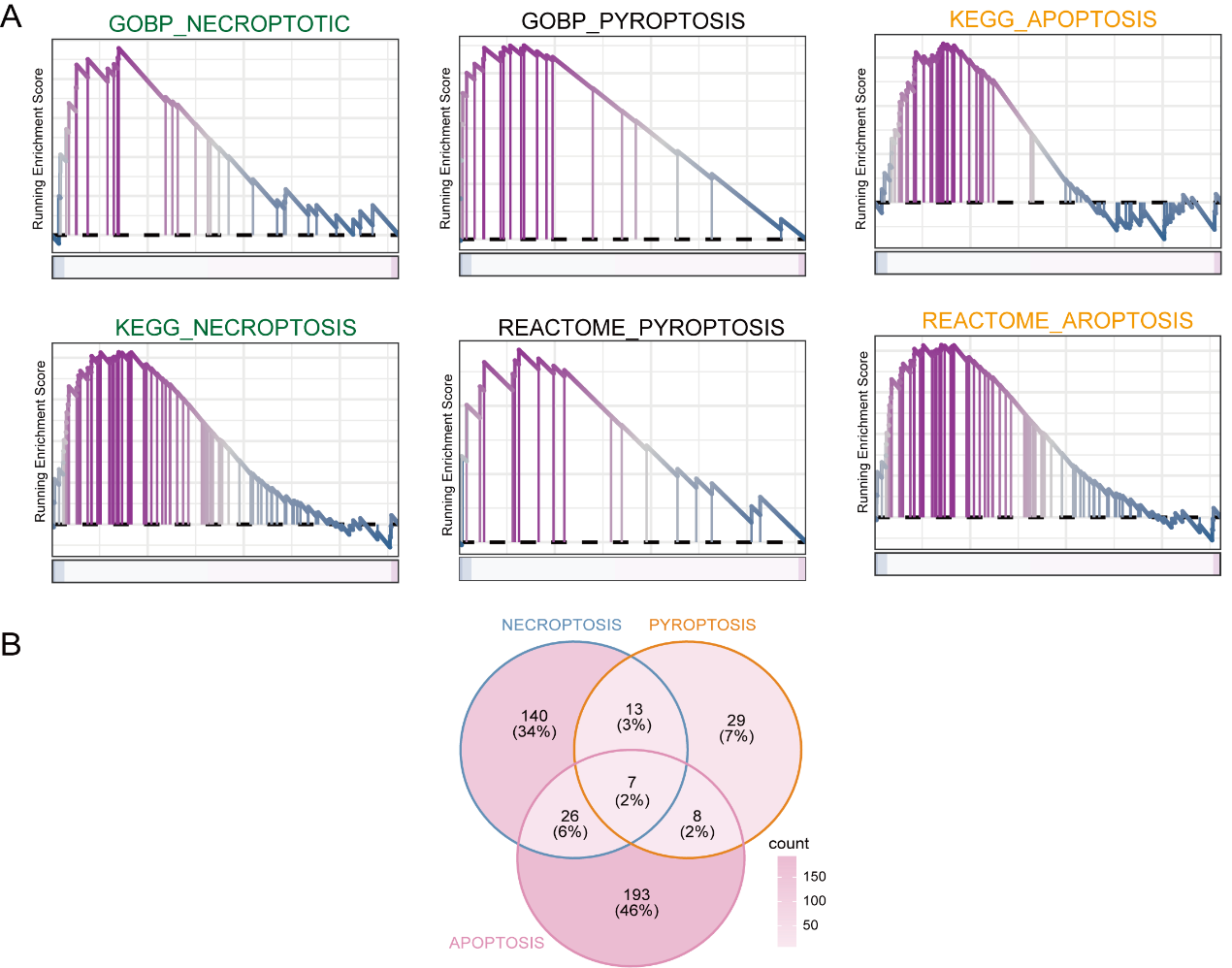


**Supplementary Figure 2** (A) GSEA enrichment plots showing significant activation of PANoptosis-related pathways, including GOBP_NECROPTOSIS, GOBP_ PYROPTOSIS, KEGG_APOPTOSIS, KEGG_NECROPTOSIS, REACTOME_ APOPTOSIS, REACTOME_PYROPTOSIS. (B) Venn diagram showing overlapping genes among apoptosis, pyroptosis, and necroptosis pathways, highlighting shared and unique components.


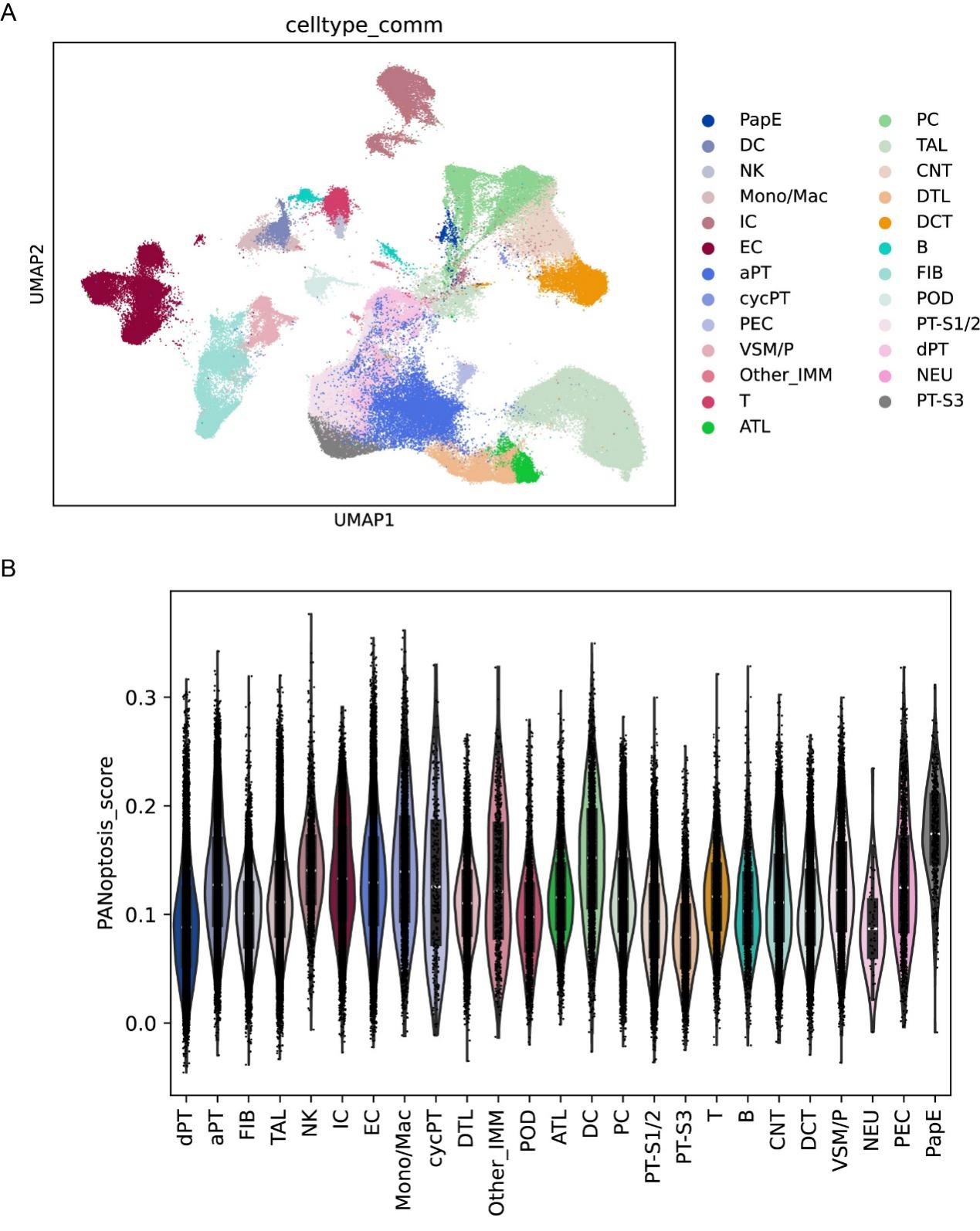


**Supplementary Figure 3** Subtype-level cell annotation and PANoptosis activity across renal and immune cell populations. (A) Uniform Manifold Approximation and Projection (UMAP) visualization of the integrated single-cell transcriptomic landscape with annotated renal parenchymal and immune cell populations. (B) Violin plots showing the distribution of PANoptosis scores across annotated cell types/subtypes; each dot represents an individual cell. Abbreviations: aPT, adaptive proximal tubule; cycPT, cycling proximal tubule; PT-S1/2, proximal tubule S1/S2; PT-S3, proximal tubule S3; dPT, distal proximal tubule; DTL, descending thin limb; DCT, distal convoluted tubule; CNT, connecting tubule; TAL, thick ascending limb; POD, podocyte; EC, endothelial cell; VSM/P, vascular smooth muscle/pericyte; FIB, fibroblast; DC, dendritic cell; NK, natural killer cell; Mono/Mac, monocyte/macrophage; Other_IMM, other immune cells.


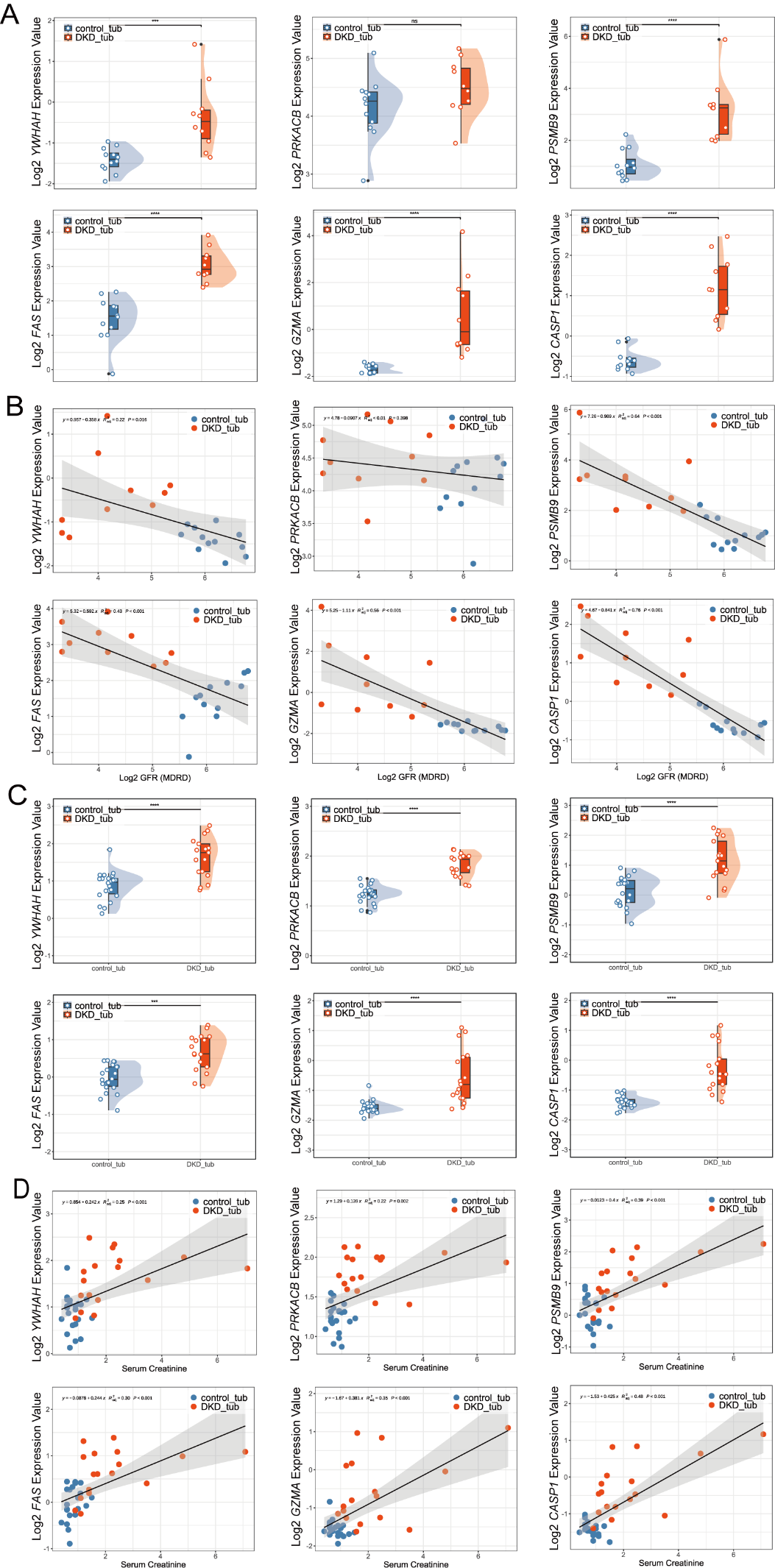


**Supplementary Figure 4** (A) Expression of PANoptosis-related genes in control and DKD tubules (Woroniecka dataset). (B) Correlation between gene expression and GFR. (C) Validation of gene expression in control and DKD tubules (Ju CKD dataset). (D) Correlation between gene expression and serum creatinine.


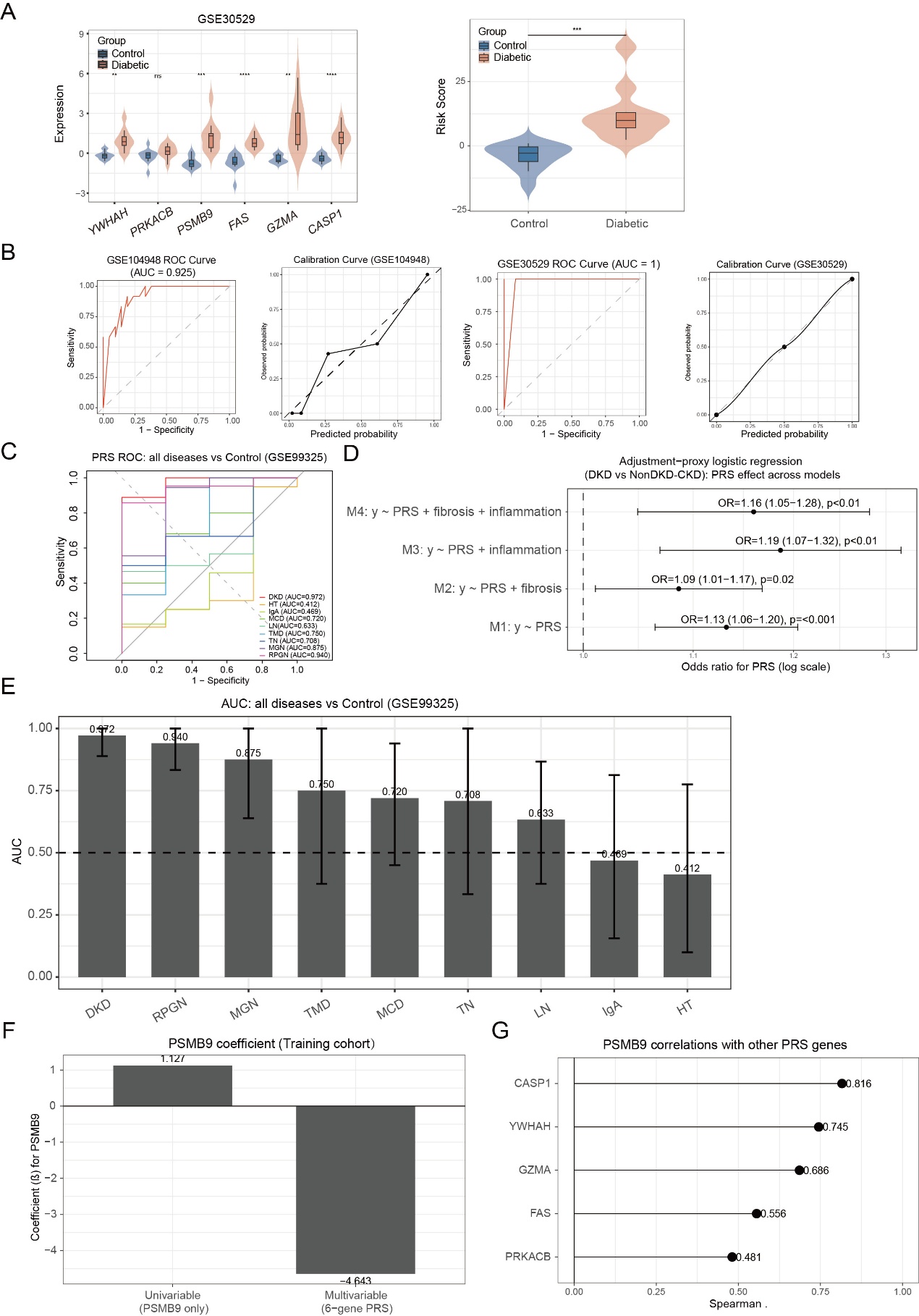


**Supplementary Figure 5** (A) Violin plots showing the expression levels of the six PRS genes (YWHAH, PRKACB, PSMB9, FAS, GZMA, CASP1) and the corresponding PRS risk scores in control and DKD samples from GSE30529. (B) ROC curve analysis evaluating the diagnostic performance of the PRS risk score in GSE30529 and GSE104948. Calibration curves of the PRS model in the validation cohorts, showing overall agreement between predicted and observed probabilities. (C) ROC curves of the PRS risk score for distinguishing diabetic kidney disease (DKD) and other chronic kidney disease (CKD) etiologies from controls in the multi-etiology cohort GSE99325, including hypertensive nephropathy (HT), IgA nephropathy (IgA), membranous glomerulonephritis (MGN), rapidly progressive glomerulonephritis (RPGN), minimal change disease (MCD), tubulointerstitial nephritis (TN), thin basement membrane disease (TMD), and lupus nephritis (LN). (D) Proxy-adjusted logistic regression analysis assessing the association between PRS and DKD versus non-DKD CKD in GSE99325, with additional adjustment for transcriptome-derived fibrosis and/or inflammation ssGSEA scores. (E) Summary of AUC values for all disease-versus-control comparisons in GSE99325, corresponding to (C). (F) PSMB9 shows an opposite coefficient direction when modeled alone versus within the six-gene PRS panel in the training cohort. (G) Spearman correlations between PSMB9 and the other PRS genes. *p < 0.05, **p < 0.01, *p < 0.001, **p < 0.0001.


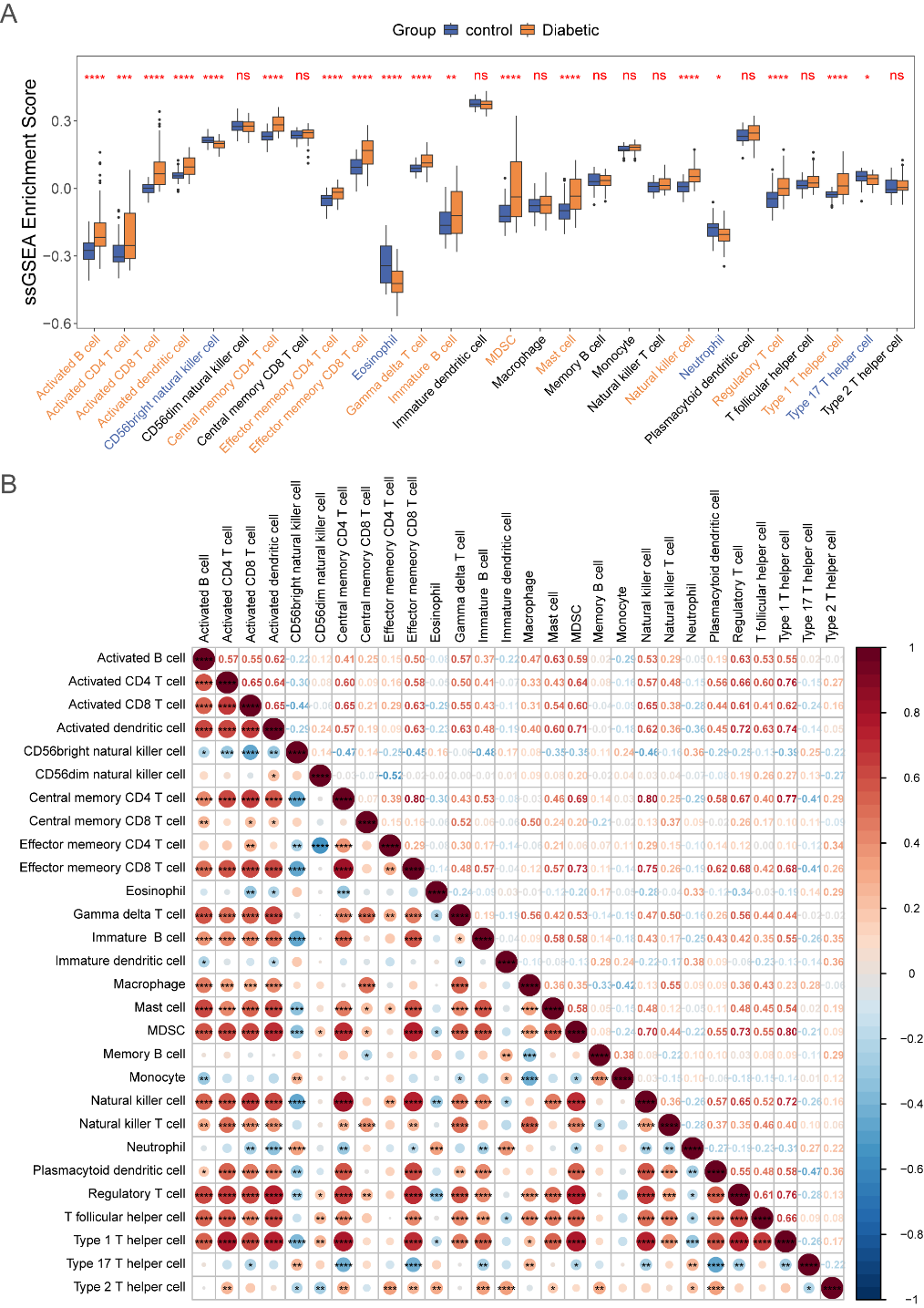


**Supplementary Figure 6** (A) Boxplots comparing immune infiltration scores between control and diabetic groups. (B) Correlation matrix of the 28 immune cell types. *p < 0.05; **p < 0.01; ***p < 0.001; ****p < 0.0001; ns, not significant.


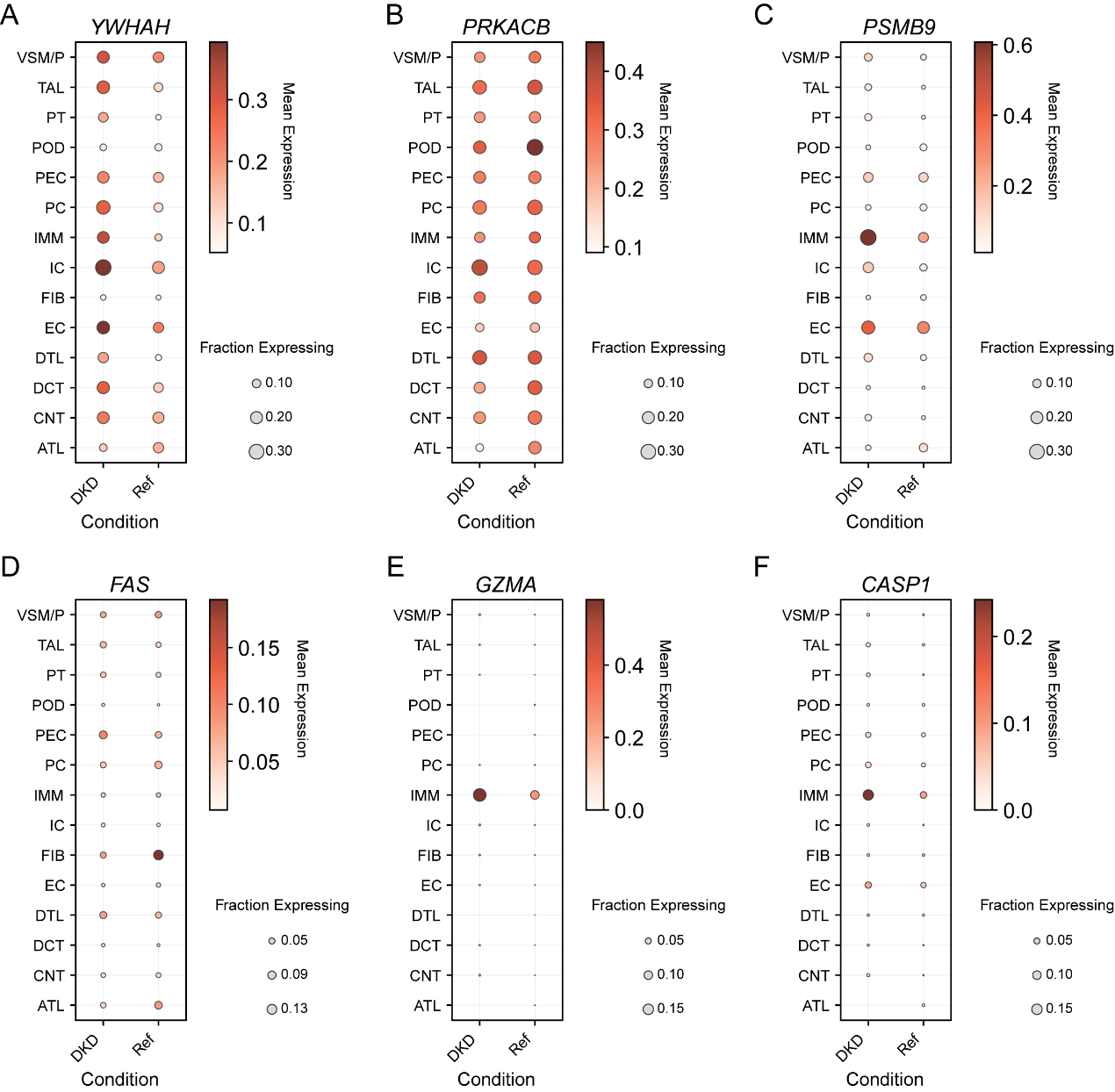


**Supplementary Figure 7** Single Nucleus RNA Sequencing of hub genes. (A) The distribution of *YWHAH*. (B) The distribution of *PRKACB*. (C) The distribution of *PSMB9*. (D) The distribution of *FAS*. (E) The distribution of *GZMA*. (F) The distribution of *CASP1*. Cell types with fewer than 1000 cells were filtered out.


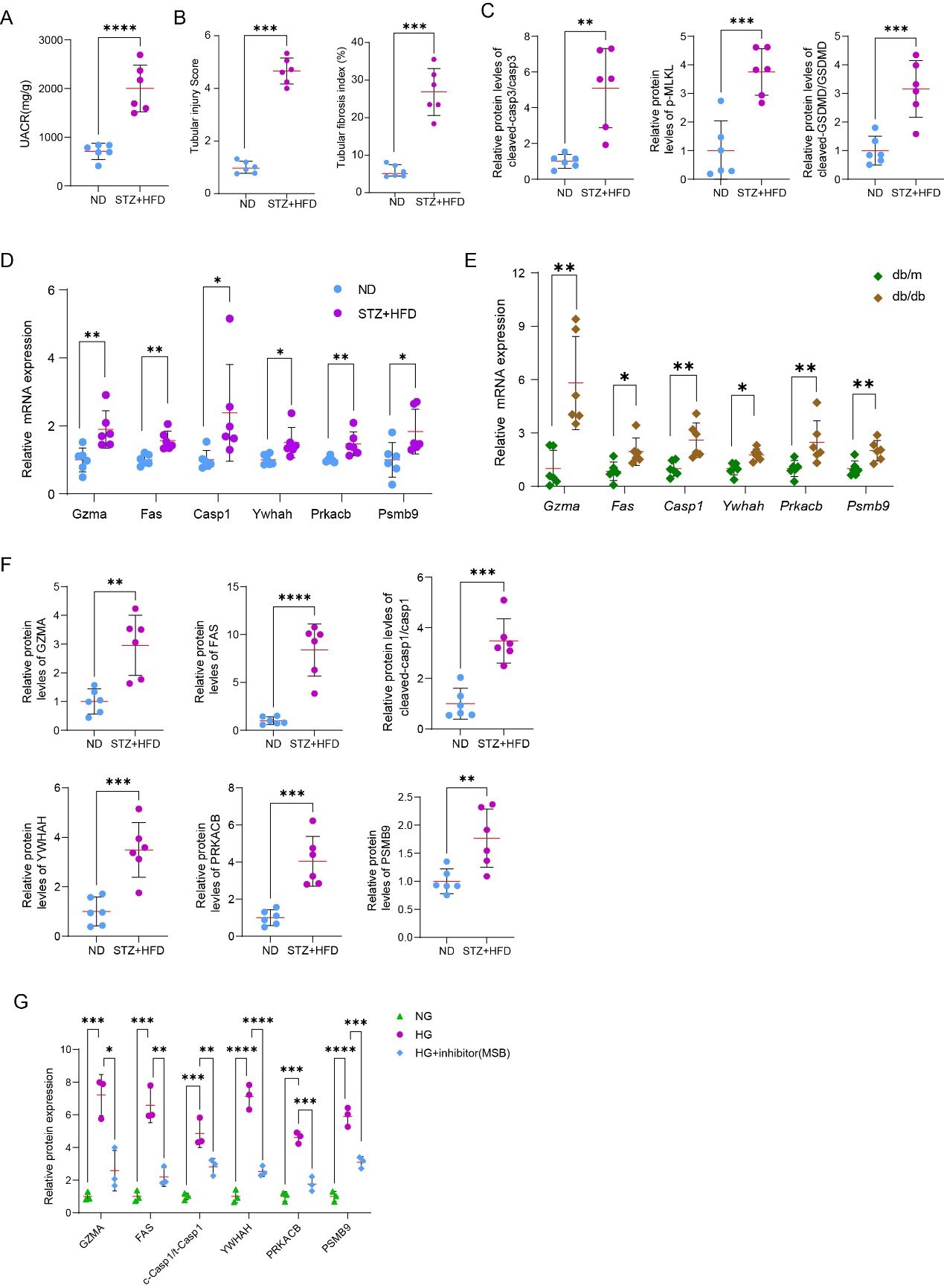


**Supplementary Figure 8** (A) Urinary albumin-to-creatinine ratio (UACR) in normal diet (ND) and streptozotocin plus high-fat diet (STZ+HFD) mice. (B) Quantification of tubular injury score and tubular fibrosis index (%). (C) Densitometric quantification of PANoptosis-associated markers in ND and STZ+HFD kidneys, including cleaved caspase-3/caspase-3, phosphorylated MLKL (p-MLKL), and cleaved GSDMD/GSDMD. (D) RT–qPCR validation of six hub PANoptosis-related genes (Gzma, Fas, Casp1, Ywhah, Prkacb, and Psmb9) in ND and STZ+HFD kidneys. (E) RT–qPCR validation of the six hub genes in db/m and db/db mouse kidneys. (F) Densitometric quantification of hub-gene proteins (GZMA, FAS, cleaved caspase-1/caspase-1, YWHAH, PRKACB, and PSMB9) in ND and STZ+HFD kidneys. (G) Relative protein expression of hub-gene products in HK-2 cells cultured under normal glucose (NG), high glucose (HG), or HG plus the PANoptosis inhibitor MSB. Each dot represents one biological replicate; data are shown as mean ± SEM. *P < 0.05; **P < 0.01; ***P < 0.001; ****P < 0.0001.


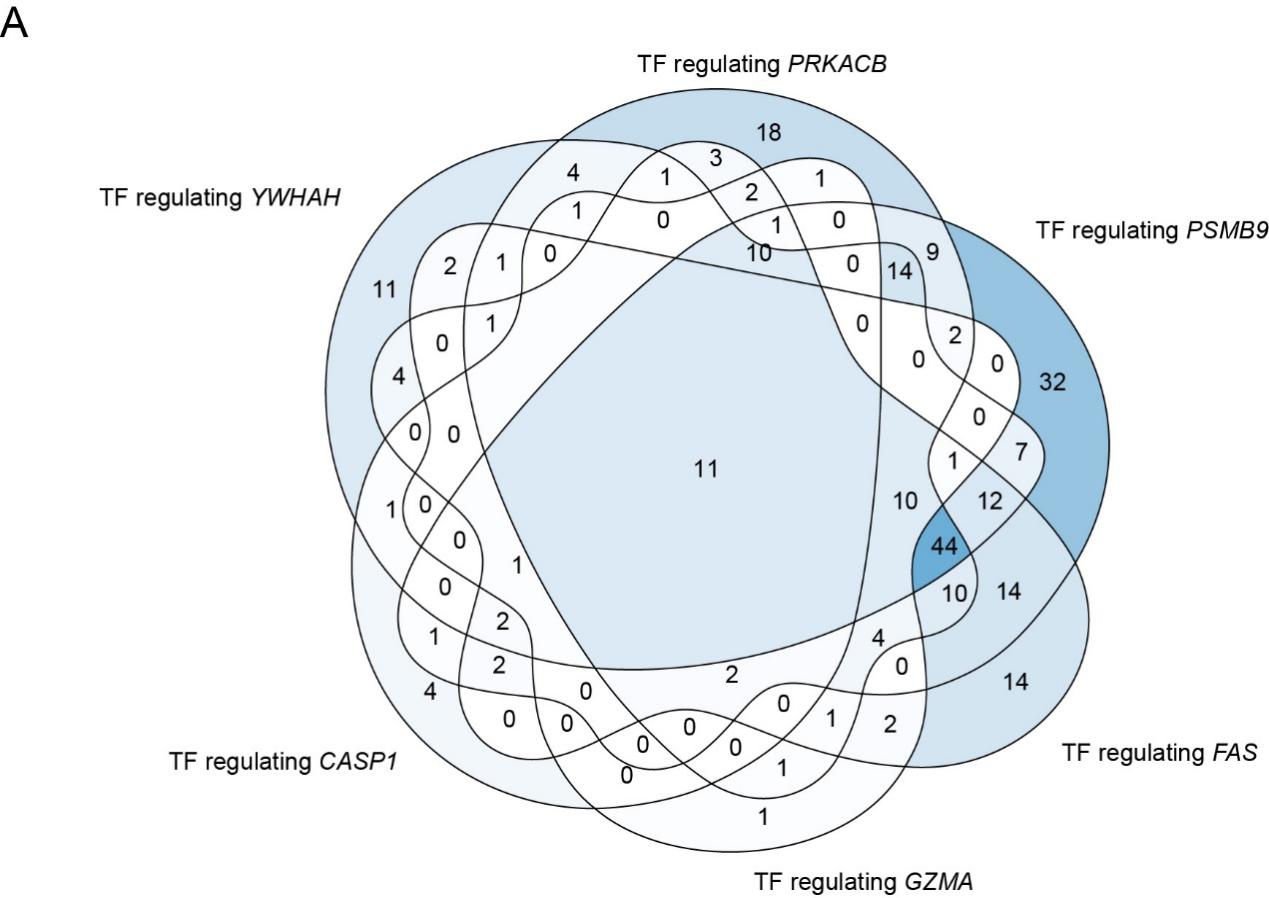


**Supplementary Figure 9** Venn diagram showing the overlap of predicted transcription factors regulating YWHAH, PRKACB, PSMB9, FAS, GZMA, and CASP1, identifying 11 common TFs shared by all six hub genes.


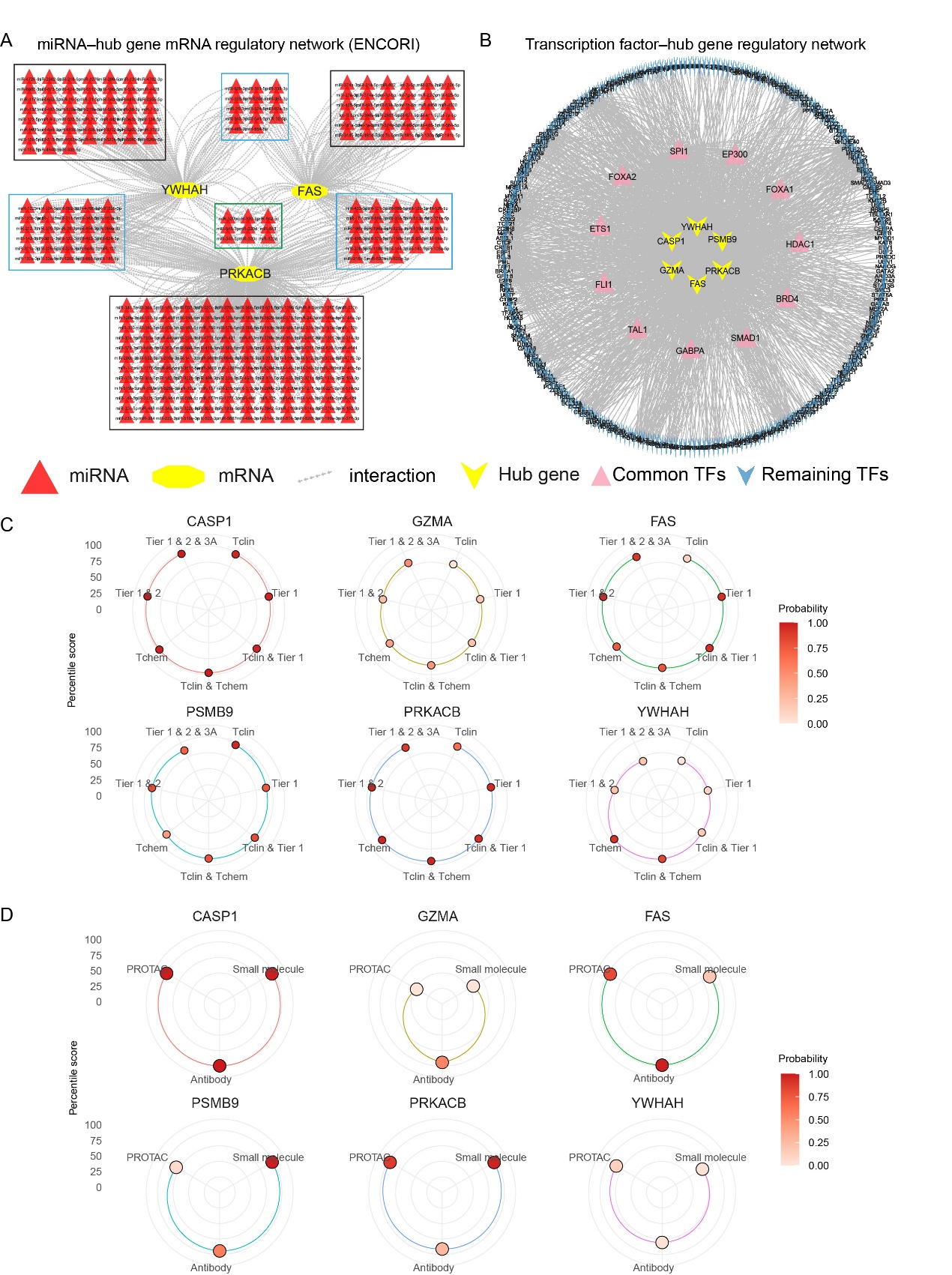


**Supplementary Figure 10** Druggability and regulatory networks of panoptosis hub genes. (A) miRNA–hub gene mRNA regulatory network for FAS, YWHAH, and PRKACB based on ENCORI-supported miRNA–mRNA pairs (Supplementary Table 4). Red triangles represent miRNAs, and yellow ovals represent hub-gene mRNAs. Dashed gray edges denote ENCORI-supported miRNA–mRNA regulatory interactions. miRNAs targeting 1, 2, or 3 hub genes are highlighted with black, blue, or green solid boxes, respectively. (B) Transcription factor (TF)–hub gene regulatory network for YWHAH, PRKACB, PSMB9, FAS, GZMA, and CASP1 derived from hTFtarget (Supplementary Table 5). Yellow inverted triangles represent hub genes. Pink triangles denote common TFs predicted to regulate all six hub genes, whereas blue triangles indicate the remaining TFs predicted to regulate at least one hub gene. Gray edges represent hTFtarget-supported TF–hub gene regulatory interactions. (C) Prediction of the druggability of panoptosis hub genes using generic DrugnomeAI models based on different druggable-gene training sets. (D) Prediction of therapeutic modalities for panoptosis hub genes using modality-specific DrugnomeAI models trained on small-molecule, antibody and PROTAC targets.
