# Supplementary material for: Identification of PANoptosis hub genes driving immune activation and tubulointerstitial injury in diabetic kidney disease by integrative bioinformatics and machine learning
Source: Front Immunol. 2026 Mar 9;17:1759781. doi: 10.3389/fimmu.2026.1759781 (PMC13006297; doi:10.3389/fimmu.2026.1759781)
Supplement: Supplementary file 3 [file Table2.docx]

Table 1

Summary of the data sets utilized in this research and their features.

| **Dataset** | **Database** | **Platform** | **Sample** | **Tissue** |
| --- | --- | --- | --- | --- |
| [GSE104954](https://www.ncbi.nlm.nih.gov/geo/query/acc.cgi?acc=GSE104954) | GEO | [GPL22945](https://www.ncbi.nlm.nih.gov/geo/query/acc.cgi?acc=GPL22945)  [GPL24120](https://www.ncbi.nlm.nih.gov/geo/query/acc.cgi?acc=GPL24120) | 17 cases of DKD and 21 cases of controls | Tubulointerstitium |
| [GSE30122](https://www.ncbi.nlm.nih.gov/geo/query/acc.cgi?acc=GSE30122) | GEO | [GPL571](https://www.ncbi.nlm.nih.gov/geo/query/acc.cgi?acc=GPL571) | 10 cases of DKD and 24 cases of controls | Tubulointerstitium |
| [GSE47185](https://www.ncbi.nlm.nih.gov/geo/query/acc.cgi?acc=GSE47185) | GEO | [GPL14663](https://www.ncbi.nlm.nih.gov/geo/query/acc.cgi?acc=GPL14663)  [GPL11670](https://www.ncbi.nlm.nih.gov/geo/query/acc.cgi?acc=GPL11670) | 18 cases of DKD and 4 cases of controls | Tubulointerstitium |
| [GSE99325](https://www.ncbi.nlm.nih.gov/geo/query/acc.cgi?acc=GSE99325) | GEO | [GPL19109](https://www.ncbi.nlm.nih.gov/geo/query/acc.cgi?acc=GPL19109)  [GPL19184](https://www.ncbi.nlm.nih.gov/geo/query/acc.cgi?acc=GPL19184) | 18 cases of DKD and 4 cases of controls | Tubulointerstitium |
| [GSE30529](https://www.ncbi.nlm.nih.gov/geo/query/acc.cgi?acc=GSE30529) | GEO | [GPL571](https://www.ncbi.nlm.nih.gov/geo/query/acc.cgi?acc=GPL571) | 10 cases of DKD and 12 cases of controls | Tubulointerstitium |
| [GSE104948](https://www.ncbi.nlm.nih.gov/geo/query/acc.cgi?acc=GSE104948) | GEO | [GPL22945](https://www.ncbi.nlm.nih.gov/geo/query/acc.cgi?acc=GPL22945)  [GPL24120](https://www.ncbi.nlm.nih.gov/geo/query/acc.cgi?acc=GPL24120) | 12 cases of DKD and 21 cases of controls | Glomerular |

Our study included six gene-expression datasets from DKD patients (five tubulointerstitial and one glomerular), as detailed in the table.
