# Supplementary material for: Identification of PANoptosis hub genes driving immune activation and tubulointerstitial injury in diabetic kidney disease by integrative bioinformatics and machine learning
Source: Front Immunol. 2026 Mar 9;17:1759781. doi: 10.3389/fimmu.2026.1759781 (PMC13006297; doi:10.3389/fimmu.2026.1759781)
Supplement: Supplementary file 5 [file Table4.docx]

**Table 3. List of primers used in this study**

| *Ywhah*-F (mouse) | GATGTCTTGGCTCTGCTTGA |
| --- | --- |
| *Ywhah* -R (mouse) | CGGTAGTAATCGCCCTTCATT |
| *Prkacb*-F (mouse) | CGGCGTGAGTGACATAAAGA |
| *Prkacb* -R (mouse) | GAGCCTCTGAACTTTGGTATGA |
| *Psmb9*-F (mouse) | CCATGGGAGGGATGCTAATTC |
| *Psmb9*-R (mouse) | TAAGCTGCGTCCACATAACC |
| *Fas*-F (mouse) | CCGAGAGTTTAAAGCTGAGGAG |
| *Fas* -R (mouse) | TTCAGGTTGGCATGGTTGA |
| *Gzma*-F (mouse) | CTGAAGGAGGCTGTGAAAGAA |
| *Gzma* -R (mouse) | CGCCAGCACAGATGGTATT |
| *Casp1*-F (mouse) | ACAAGATCCTGAGGGCAAAG |
| *Casp1* -R (mouse) | CCTGATTCAGCACTCTCTTCTC |
| *Gapdh*-F (mouse) | GCAAATTCAACGGCACAGTCAAG |
| *Gapdh*-R (mouse) | TCGCTCCTGGAAGATGGTGATG |
