# Supplementary material for: Identification of PANoptosis hub genes driving immune activation and tubulointerstitial injury in diabetic kidney disease by integrative bioinformatics and machine learning
Source: Front Immunol. 2026 Mar 9;17:1759781. doi: 10.3389/fimmu.2026.1759781 (PMC13006297; doi:10.3389/fimmu.2026.1759781)
Supplement: Supplementary file 8 [file Table7.docx]

Data availability. The single-cell RNA-seq datasets analyzed in this study were obtained from the CZ CELLxGENE portal (https://cellxgene.cziscience.com/)

Code availability. The scripts used for the main analyses in this study are publicly available at <https://github.com/yt9805/Code/> and are also provided as Supplementary Material 7. For single-cell preprocessing/annotation and cell–cell communication analyses, our implementation was developed with reference to the workflow and example scripts from the KPMP Cell State Atlas repository (https://github.com/KPMP/Cell-State-Atlas-2022)
