# Supplementary figures and images for: Identification of PANoptosis hub genes driving immune activation and tubulointerstitial injury in diabetic kidney disease by integrative bioinformatics and machine learning
Source: Front Immunol. 2026 Mar 9;17:1759781. doi: 10.3389/fimmu.2026.1759781 (PMC13006297; doi:10.3389/fimmu.2026.1759781)

Workflow for hub-gene selection and PRS construction/validation


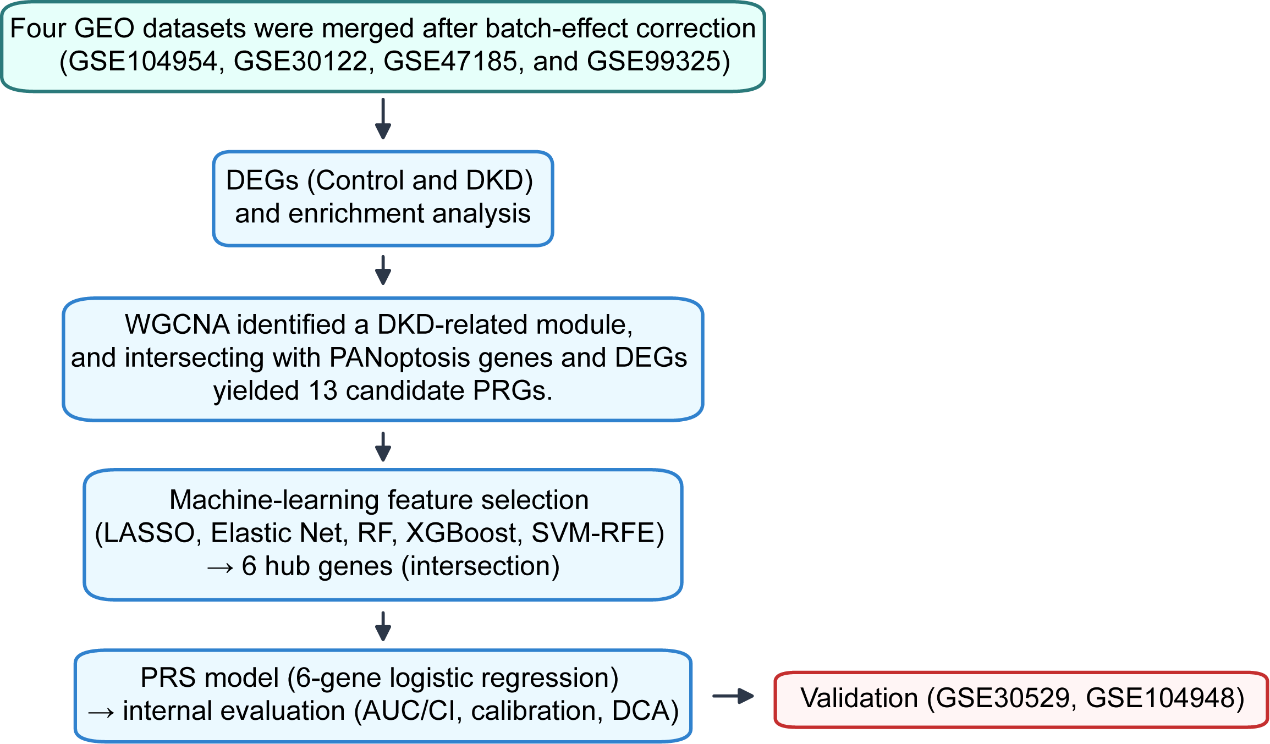

Supplement: Supplementary file 9 [file Table8.docx]
